# Generation of Cascades of Care for Diabetes and Hypertension Care Continuum in Cambodia: Protocol for a Population-Based Survey Protocol

**DOI:** 10.2196/36747

**Published:** 2022-09-02

**Authors:** Vannarath Te, Edwin Wouters, Veerle Buffel, Wim Van Damme, Josefien van Olmen, Por Ir

**Affiliations:** 1 Health Policy and Systems Research Unit National Institute of Public Health Phnom Penh Cambodia; 2 Health Policy Unit Department of Public Health Institute of Tropical Medicine (Antwerp) Antwerp Belgium; 3 Quality of Integrated Care Spearhead Research Public Health & Primary Care The University of Antwerp Antwerp Belgium; 4 Center for Longitudinal & Life Course Studies Department of Sociology The University of Antwerp Antwerp Belgium

**Keywords:** diabetes, hypertension, cascade of care, implementation research, care models, population-based survey, continuum of care

## Abstract

**Background:**

Cardiovascular diseases (CVDs) were accountable for 24% of the total deaths in Cambodia, one of the low- and middle-income countries, where primary health care (PHC) settings generally do not perform well in the early detection, diagnosis, and monitoring of leading risk factors for CVDs, that is, type 2 diabetes (T2D) and hypertension (HT). Integrated care for T2D and HT in the Cambodian PHC system remains limited, with more than two-thirds of the population never having had their blood glucose measured and more than half of the population with T2D having not received treatment, with only few of them achieving recommended treatment targets. With regard to care for T2D and HT in the public health care system, 3 care models are being scaled up, including (1) a hospital-based model, (2) a health center–based model, and (3) a community-based model. These 3 care models are implemented in isolation with relatively little interaction between each other. The question arises as to what extent the 3 care models have performed in providing care to patients with T2D or HT or both in Cambodia.

**Objective:**

This protocol aims to show how to use primary data from a population-based survey to generate data for the cascades of care to assess the continuum of care for T2D and HT across different care models.

**Methods:**

We adapt the HIV test-treat-retain cascade of care to assess the continuum of care for patients living with T2D and HT. The cascade-of-care approach outlines the sequential steps in long-term care: testing, diagnosis, linkage with care, retention in care, adherence to treatment, and reaching treatment targets. Five operational districts (ODs) in different provinces will be purposefully selected out of 103 ODs across the country. The population-based survey will follow a multistage stratified random cluster sampling, with expected recruitment of 5280 eligible individuals aged 40 and over as the total sample size. Data collection process will follow the STEPS (STEPwise approach to NCD risk factor surveillance) survey approach, with modification of the sequence of the steps to adapt the data collection to the study context. Data collection involves 3 main steps: (1) structured interviews with questionnaires, (2) anthropometric measurements, and (3) biochemical measurements.

**Results:**

As of December 2021, the recruitment process was completed, with 5072 eligible individuals participating in the data collection; however, data analysis is pending. Results are expected to be fully available in mid-2022.

**Conclusions:**

The cascade of care will allow us to identify leakages in the system as well as the unmet need for care. Identifying gaps in the health system is vital to improve efficiency and effectiveness of its performance. This study protocol and its expected results will help implementers and policy makers to assess scale-up and adapt strategies for T2D and HT care in Cambodia.

**Trial Registration:**

International Standard Randomised Controlled Trials Number (ISRCTN) registry ISRCTN41932064; https://www.isrctn.com/ISRCTN41932064

**International Registered Report Identifier (IRRID):**

DERR1-10.2196/36747

## Introduction

Globally, cardiovascular diseases (CVDs) are responsible for the death of 17.9 million people annually, accounting for 31% of all deaths [1]. More than 75% of deaths attributable to CVDs occur in low- and middle-income countries (LMICs), where primary health care (PHC) settings generally do not perform well in the early detection, diagnosis, and monitoring of type 2 diabetes (T2D) and hypertension (HT), which are the leading risk factors for CVDs [1,2]. In regions where early diagnosis and care are not available or inadequate, T2D and HT-related complications—including CVDs, kidney disease, neuropathy, blindness, and lower-extremity amputation—are a significant cause of morbidity and mortality among people with T2D or HT or both [3,4]. The resulting complications will increase health care costs and pose challenges to population health, socioeconomic development, and health systems [5,6], negatively affecting country’s effort to achieve universal health coverage [7]. Globally, adult populations with HT and T2D had increased from 594 million to 1.13 billion between 1975 and 2015 [8] and from 4.7% to 8.5% between 1980 and 2014 (with approximately 422 million living with T2D in 2014), respectively [2]. Access to a lifelong continuum of care is therefore critical for those living with T2D or HT or both as well as for the prevention of CVDs [9]. The World Health Organization Package of Essential Noncommunicable Disease Interventions (WHO PEN) offers substantial international support for PHC services to include care for T2D and HT in LMICs [10].

In Cambodia, CVDs were estimated to account for 24% of the total deaths in 2018 [11], and in 2016 the prevalence rates of T2D and HT were 9.6% and 14.2%, respectively, among adult population between the ages of 18 and 69 years [12]. This seems a significant increase, as the prevalence of T2D between ages 25 and 64 was only 2.9% in 2010 [12]. However, integrated care for T2D and HT in the Cambodian PHC system remains limited [13]. More than two-thirds of the population have never had their blood glucose measured, and more than half of the population with T2D is not receiving treatment [12,14]. The proportion of patients with T2D accessing treatment is low, with few achieving recommended treatment targets [15].

The response to these T2D and HT epidemics requires concerted effort from both global health governance and Cambodia’s health system. Cambodia has a pluralistic health system, with a public health care system operated by the Ministry of Health, complemented by many private health care services that mainly offer outpatient curative care, operating largely without sufficient steering and coordination from the government [16]. The government’s public health care system was established based on a district health system model, following the PHC approach. With regard to the care for T2D and HT in the public health care system, the following 3 care models are being scaled up: (1) a *hospital-based model*, (2) a *health center–based model*, and (3) a *community-based model*.

The hospital-based model is a standard care model for T2D and HT that is available at district or provincial referral hospitals as part of outpatient consultation. These referral hospitals provide ambulatory care and support the health centers in treating serious cases. Health centers are allowed to take care of mild or stable cases without complications. The referral hospitals will treat serious cases [13]. In 2018, the Ministry of Health added a second component to this standard care: 29 district and provincial referral hospitals (out of 117) provided exclusive health care services for patients with T2D or HT or both in a separate section, giving explicit attention to these conditions [17].

The health center–based model has been given increasing attention by the Ministry of Health with support from the World Health Organization through the adoption of the WHO PEN for PHC [10]. The National Standard Operating Procedure for T2D and HT Management in Primary Care was developed out of the WHO PEN and approved in 2019 to strengthen implementation of the integrated basic care for T2D and HT in the PHC system. In this health center–based model, health center staff are trained to do screening, provide follow-up care for patients with T2D or HT or both with mild and stable conditions (with diagnosis only undertaken at the referral hospital), and offer health education and counseling on healthy behavior as part of screening for CVD risk factors [13]. With mild HT cases, health center staff are allowed to initiate treatment. At the health center level, care for both T2D and HT is described in a national clinical guideline on the minimum package of activities specified for health centers [18]. Yet, in practice, implementation of this guideline is not as complete as intended because the public health care system has not yet been substantially reoriented from primarily addressing acute health needs toward continuing care for chronic conditions. The public health system currently focuses on communicable diseases (HIV and AIDS, tuberculosis, malaria, diarrhea, and respiratory diseases) and maternal and child health [19]. In early 2020, only 86 health centers (out of 1221) implemented the WHO PEN program since its pilot in 2015 [17].

The community-based model is predominantly run by a Cambodian nongovernmental organization called MoPoTsyo that operates Peer Educator Networks with 4 main key services for patients with T2D or HT or both. These services include (1) self-management training through peer educator visits, (2) laboratory tests, (3) physician consultations, and (4) low-cost medicines delivered through a revolving drug fund program to the members in the network in 8 out of 24 provinces across the country. By 2019, 255 peer educators have been trained to serve over 40,000 patients [20]. In this community-based model, peer educators, who are patients with T2D or HT or both themselves, have been trained by MoPoTsyo to be educators and counselors on lifestyle change. Peer educators also assist registered patients in the networks to have access to professional medical consultations at the public referral hospitals with which they have partnership agreements [20].

These 3 care models are implemented in isolation with relatively little interaction between each other. There have been few empirical studies on their performance. The question arises as to what extent the 3 care models have performed in providing care to people with T2D or HT or both in Cambodia. Care models that are integrated in terms of shared information and resource coordination have shown to be effective and efficient in many contexts [21]; however, implementation and scale-up of effective care models for T2D and HT remain limited, especially in LMICs. How well different models perform in contributing to good health outcomes is also not well documented. The outcomes of chronic care are difficult to measure, as such care does not have a clear end point, but requires comprehensive illness management along a continuum of care, from detection and diagnosis for initiating treatment and follow-up to successful management of the illness. This complexity requires a comprehensive framework of measurement to assess the performance of care for T2D and HT.

Inspired by noticeable successes in providing the continuum of care to people living with HIV in Cambodia [22], we adapted the HIV test-treat-retain cascade of care [23] in this study protocol to assess the continuum of care for patients living with T2D or HT or both. This method documents how many patients are lost to follow-up between the stages of testing and diagnosis, linkage with and retention in care, and adherence to treatment and control of health conditions. The cascade-of-care approach outlines the aforementioned sequential steps in long-term care. Recently, this approach has been applied to T2D and HT by pooling secondary data from cross-sectional studies of nationally representative surveys in LMICs [24,25]. This was used to produce cascades of care as an approach to assess the performance of health systems to meet the continuum of care for patients living with T2D or HT or both. Two studies, one in the United States and the other in South Africa, developed and field tested the cascade of care for T2D and HT [26,27]. Their analysis was mainly based on extracted secondary data from broader nationally representative surveys, not specifically designed for this purpose.

Given that Cambodia implements T2D and HT services through 3 different care models, we propose the cascade-of-care approach to assess the performance of these care models along the continuum of care. We will do so using primary data collection. This study protocol aims to serve as a tool to generate the cascades of care for T2D and HT for the 3 care models using the primary data of a population-based survey. The specific aims are as follows:

- To generate the cascades of care for T2D for (1) hospital-based care, (2) health center–based care (WHO PEN), (3) community-based care (Peer Educator Network), and (4) coexistence of 1, 2, and 3;
- To generate the cascades of care for HT for (1) hospital-based care, (2) health center–based care (WHO PEN), (3) community-based care (Peer Educator Network), and (4) coexistence of 1, 2, and 3;
- To compare the cascades of care for T2D between the care models 1-4;
- To compare the cascades of care for HT between the care models 1-4.

## Methods

### Study Design

This study protocol is part of a larger population-based survey—the SCUBY (Scale up diabetes and hypertension care for vulnerable people in Cambodia, Slovenia and Belgium) project [28], which includes other substudies focusing on (1) the health status of people aged 40 and over and the existence of comorbidities, (2) health care utilization and health care expenditure among people aged 40 and above and people living with T2D or HT or both, (3) the lifestyle and knowledge of T2D and HT among people living with T2D or HT or both, and (4) the self-management and social support for people living with T2D or HT or both.

To meet the aforesaid specific aims, 5 operational districts (ODs) in different provinces will be purposefully selected out of 103 ODs across the country. The selection is based on a mapping exercise conducted in the SCUBY project. Only in the OD Daunkeo the 3 care models coexist. The OD Samrong in a bordering province hosts a typical noncommunicable disease (NCD) clinic at the referral hospital (the WHO PEN and Peer Educator Network are not there yet). The OD Kong Pisey does not have the WHO PEN and the NCD clinic at the referral hospital (only the Peer Educator Network of MoPoTsyo exists—with relatively strong network). The OD Pearaing is one of the ODs piloting the WHO PEN and started implementing the program since 2015. At the time of study, 8 of 9 health centers have implemented the WHO PEN. The OD Sotr Nikum is historically and significantly influenced by the financial aid of various development partners and nongovernmental organizations—contextual factor is a focus. The referral hospital in this OD has a Chronic Disease Clinic, where people with T2D or HT or both and those with HIV seek treatment and care [29]. At the time of study, 5 of 25 health centers have implemented the WHO PEN. Table 1 shows the existence of care provision for T2D and HT in each OD.

**Table 1:** Selected provinces and ODs^a^ with different types of care models.

OD name	Province	Existing care provision	Care model
Samrong	Oddar Meanchey	NCD^b^ clinic at the referral hospital	Hospital-based care
Pearaing	Prey Veng	NCD clinic^c^ + WHO PEN^d^ (high coverage)	Health center–based care
Sotr Nikum	Siem Reap	NCD clinic^c^ + WHO PEN (low coverage)	Health center–based care with context
Kong Pisey	Kampong Speu	Peer Educator Network^e^	Community-based care
Daunkeo	Takeo	NCD clinic + WHO PEN + Peer Educator Network	Coexistence of care

^a^OD: operational district.

^b^NCD: noncommunicable disease.

^c^In the WHO PEN implementation arrangement, the referral hospital in the OD supports the health centers in providing the secondary care.

^d^WHO PEN: World Health Organization Package of Essential Noncommunicable Disease Interventions.

^e^The Peer Educator Network arranges a medical consultation for their registered patients once a week at the referral hospital in the OD.

This population-based survey includes 2 questionnaires (Multimedia Appendices 1 and 2). One questionnaire is directed at household heads and inquires about the household’s socioeconomic status, member characteristics, general health of the household members, and their access to health care and health-related expenditure. The other questionnaire is for eligible adults (ie, adults aged ≥40) of the selected households and inquires about their sociodemographic information, health status and comorbidity, quality of life, health care utilization, social support, behavioral measurements, and knowledge of T2D and HT for known patients with T2D or HT or both in particular. Known patients with T2D or HT or both are also asked about their medical adherence and decision-making power over diet.

### Sampling and Sample Size

This population-based survey follows a multistage stratified random cluster sampling [30]. For the sampling procedure, each OD is considered as a stratum due to the care model present (Table 1). Based on rules of stratification, each stratum is theoretically independent from one another, and its selection can be based on the aforementioned specific aims [30]. In each stratum, clusters of primary sampling units (villages) will be randomly sampled.

**Figure 1:**
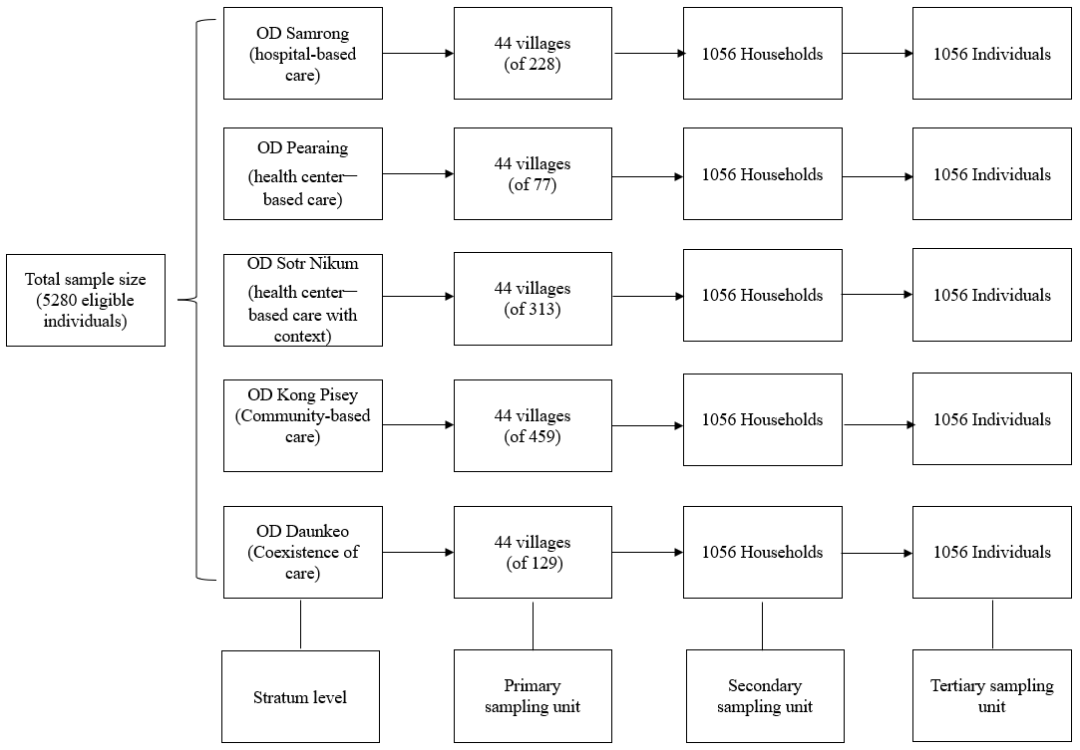
Flow of the sampling frame.

Based on the multistage stratified random cluster sampling, 3 stages of stratification are applied, and in each stage randomization is employed. ODs are the strata, and health centers impacted by the types of care models are selected. For the first stage of stratification, sampling with equal probability (equal probability selection method) will be used to determine the number of villages under the catchment areas of the impacted health centers in each OD. This equal selection is also made to ensure oversampling for some ODs. If using proportionate allocation of the sample units across the ODs, the sample selected from the ODs representing certain types of care models would be too low to have enough statistical power for the analyses. In this survey, disproportionate allocation is done to randomly select equal-sized samples in the 5 ODs [30]. For the second stage of stratification, households having adult(s) aged 40 and above (secondary sampling units) in the selected villages are selected and listed. Systematic random sampling is used to select the households from each village. For the third stage of stratification, only 1 individual meeting the eligibility criteria (described later) is randomly selected from each household to reduce the clustering effect. Thus, 1 eligible individual is selected from each household, making the total sample size of eligible individuals the same as the sample size of households (Figure 1).

The sample size of households in all the selected 5 ODs is calculated based on the following formula [30]:

n_h_ = (z^2^)(r)(1–r)(f)(k)/(p)(*γ*)(e^2^)

where n_h_ is the parameter to be calculated and the sample size in terms of the number of households to be selected; z is the statistic that defines the level of confidence desired (1.96 for the 95% level of confidence); r is an estimate of a key indicator to be measured by the survey (the key indicator being T2D prevalence—this r being 0.1 according to 10% of T2D prevalence among adults aged 40 and over—the national STEPS survey 2016 [12]); f is the sample design effect (1.5 used in accordance with the national STEPS survey 2016 [12] and the Cambodian demography and health survey 2014 [19]); k is a multiplier to account for the anticipated rate of nonresponse (1.2 for 20% anticipated rate, as used in the national STEPS survey 2016 [12]); p is the proportion of the total population accounted for by the target population and upon which the parameter r is based (0.24 for 24% [31]); *γ* is the average household size (4.6 for the number of persons per household in the selected provinces—census 2019 [32]); and e is the margin of error to be attained (0.01 for the level of precision at 10% of r).

While HT prevalence is higher, T2D prevalence of 10% [12] is used as the main key indicator of interest to determine the sample size, which is important for the cascade of care. Based on the aforementioned formula, the total sample size would be 5637 households, including 20% of anticipated nonresponse rate. Enlarging the sample size to include enough patients to assure significant differences for the detection of the number of patients with T2D having blood glucose under control would increase the budget 5-fold, which is not feasible. Taking feasibility and budget constraint into consideration, a fixed cluster size of 24 households per village, with 44 villages randomly selected in each OD, will be applied for the sake of controlling the total sample size and interviewer workloads [30]. Thus, a cluster size of 24 households per village over the total villages of 220 would yield the total sample size of 5280 households (also equal 5280 eligible individuals).

### Target Population and Recruitment Strategy

Adults aged 40 and above are the target population. This age group is appropriate for screening for T2D and HT according to the national standard operating procedure for T2D and HT management in primary care [13]. Other recruitment criteria include (1) being usual members of the household, having stayed in the household the night before the interview or not been absent for more than 6 months; (2) being physically and mentally capable of answering the questions; and (3) providing consent to participate in the study.

The starting point of recruitment is a list of all the eligible households in the selected villages (Multimedia Appendix 3). The list will be constructed by a listing team with support of a local authority, listing households in the selected village having at least one adult aged 40 and above. When a household is selected, 1 household member aged 40 and above will be selected for inclusion in the study. If the selected eligible individual is not present in the household during the first-time visit, 2 repeated callbacks and follow-up will be applied. Only if all these attempts fail, the selected participants would be replaced: the respective households would be replaced with the next household in a row of the eligible household list constructed. The replacement household would be selected following the procedure described earlier. If the eligible individuals can be contacted but express refusal to participate in the study after a few times of failed explanation, the individuals as well as the households would be dropped from the study.

### Data Collection Procedure

Data collection process will follow the STEPS survey approach [33], with modification of the sequence of the steps to adapt the data collection to the study context. There are 3 main steps of data collection: (1) structured interviews with questionnaires, (2) anthropometric measurements, and (3) biochemical measurements. The modification will entail data collectors taking anthropometric measurements and biochemical measurements of the eligible individuals before administering the 2 sets of questionnaires.

The anthropometric measurements include measurements of blood pressure, body weight, height, and waist and hip circumferences. For blood pressure measurements, participants will rest at least 15 minutes prior to the measurement and 3 readings will be taken 3 minutes apart from one another, with the left arm recommended for the measurement [33]. For the biochemical measurements, testing of fasting blood glucose (FBG) will be carried out for all the participants (Multimedia Appendix 4) and glycated hemoglobin A_1c_ (HbA_1c_) and creatinine for known participants with T2D or participants having FBG of 126 mg/dl or more (Multimedia Appendix 5). Data will be digitally collected using the KoBoToolbox system developed by the Harvard Humanitarian Initiative [34]*.*

For the point-of-care measurement of FBG (capillary plasma value), the On Call Plus (ACON USA), which is compliant with the US Food and Drug Administration regulations [35], will be used. It is widely used in the WHO PEN program in Cambodia. The HemoCue HbA_1c_ 501 System, whose quality is ensured by the International Federation of Clinical Chemistry and Laboratory Medicine and the National Glycohemoglobin Standardization Program [36], will be employed as a point-of-care test for HbA_1c_. Regarding the anthropometric measurement, Omron JPN500, which is clinically validated by the Association for the Advancement of Medical Instrumentation and European Society of Hypertension [37], will be used to measure blood pressure. A flat weight scale (Seca-803), height measuring system (Seca-217), and ergonomic circumference measuring tape with extra waist-to-hip-ratio calculator (Seca-203) will be used to measure weight, height, and waist and hip circumferences of the participants, respectively. Seca is internationally recognized as producing highly accurate scales equipped with high-precision measuring technology [38].

### Statistical Analysis Plan

For the analysis plan, 6 cascade bars, as explained in Tables 2 and 3, will be used to generate the cascades of care for T2D and HT, respectively. A fixed denominator approach will be followed as it enables readers to see the leakages between stages of the continuum of care [39]. The denominator is the total number of eligible individuals aged 40 and above having T2D (for the T2D cascade of care) and HT (for the HT cascade of care). We will produce the cascade of care for T2D and HT for the selected ODs hosting different existing care models. These cascades of care—in essence, a series of bar charts—will subsequently be translated into cumulative probabilities. The bivariate analysis will be used to identify potential factors associated with the outcome variables—prevalence, testing, diagnosis, in care, in treatment, and under control bars. At the initial stage, the chi-square test will be used to determine the association between explanatory variables and outcome variables. The explanatory variables will include participants’ age, sex, marital status, educational level, household wealth quintile, health care utilization, and care model setting. Variables with statistically significant level (*P*<.2) will be included in a multiple logistic regression model. In addition to the aforementioned variables, BMI, lifestyle, knowledge of T2D and HT, self-management, and social support will be included in the multiple logistic regression model for the outcome variables—in care, in treatment, and under control bars [24,25]. In the multiple logistic regression model, the backward elimination method will be used. The process will start with all the identified explanatory variables. Then, variables with the highest *P* value will be eliminated from the model one by one at a time. The process will be repeated until all the variables in the model are statistically significant with a cut-off point of *P* value <.05. This knowledge will allow us to identify which characteristics stimulate the probability of not reaching the next step in the cascade of care, thereby identifying patient groups not adequately reached.

**Table 2:** Defined groups of participants for each bar of the cascade of care for T2D^a^.

Bars of the cascade of care for T2D	Definitions	Questions extracted for analysis
1. Prevalence of the target population living with T2D	Participants having biochemical measurement of FBG^b^ (capillary plasma value) ≥126 mg/dl (7 mmol/L) and HbA_1c_^c^ level ≥6.5% [24,40,41]	Measurement of FBG Measurement of HbA_1c_
		Have you ever been told by a doctor or other health worker that you have T2D?
	or Participants reporting use of drugs for T2D, irrespective of their biomarker values	
2. Number of the target population with T2D ever tested for T2D	Classified patients with T2D having had FBG tested in the last 3 years	Have you ever had your blood glucose tested in the last 3 years?
3. Number of those tested ever diagnosed for T2D	Tested patients with T2D reporting ever being told by a doctor or other health worker as having T2D	Have you ever been told by a doctor or other health worker that you have T2D?
4. Number of those diagnosed in care	Diagnosed patients with T2D reporting getting treatment/care for their conditions at least once in the past 12 months	Did you get treatment/care for your T2D condition in the past 12 months?
5. Number of those in care receiving treatment	In-care patients with T2D reporting using drugs for T2D or insulin in the past 2 weeks or in-care patients with T2D reporting following advice to lose weight, stop smoking, do physical exercise, and be on special prescribed diet	Are you currently receiving any of the following treatment/advice for your T2D condition prescribed by a doctor or health care worker? Insulin or drugs (medication) that you have taken in the past 2 weeks Are you currently receiving any of the following treatment/advice for your T2D condition prescribed by a doctor or health care worker? Special prescribed diet and advice to lose weight and advice to stop smoking and advice to start or do more physical exercise
6. Number of those receiving treatment being under control	In-treatment patients with T2D having HbA_1c_ level <8% [24]	Measurement of HbA_1c_ for the known T2D

^a^T2D: type 2 diabetes.

^b^FBG: fasting blood glucose.

^c^HbA_1c_: glycated hemoglobin A_1c_.

**Table 3:** Defined groups of participants for each bar of the cascade of care for HT^a^.

Bars of the cascade of care for HT	Definitions	Questions extracted for analysis
1. Prevalence of the target population living with HT	Participants having systolic blood pressure ≥140 mmHg or diastolic blood pressure ≥90 mmHg [12] or	Measurement of blood pressure (mean of the second and third readings)
	Participants reporting use of drugs for HT, irrespective of their blood pressure values	Have you ever been told by a doctor or other health worker that you have HT?
2. Number of the target population with HT ever tested for HT	Classified patients with HT having had a blood pressure measured in the last 3 years	Have you ever had your blood pressure measured in the last 3 years?
3. Number of those tested ever diagnosed for HT	Tested patients with HT reporting ever being told by a doctor or other health worker as having HT	Have you ever been told by a doctor or other health worker that you have HT?
4. Number of those diagnosed in care	Diagnosed patients with HT reporting getting treatment/care for their conditions at least once in the past 12 months	Did you get treatment/care for your HT condition in the past 12 months?
5. Number of those in care receiving treatment	In-care patients with HT reporting using drugs for HT in the past 2 weeks or	Are you currently receiving any of the following treatment/advice for your HT condition prescribed by a doctor or other health worker? Drugs (medication) that you have taken in the past 2 weeks
	In-care patients with HT reporting following advice to lose weight, stop smoking, do physical exercise, and reduce salt intake	Are you currently receiving any of the following treatment/advice for your HT condition prescribed by a doctor or other health worker? Advice to reduce salt intake and advice to lose weight and advice to stop smoking and advice to start or do more physical exercise
6. Number of those receiving treatment being under control	In-treatment patients with HT having systolic blood pressure <140 mmHg and diastolic blood pressure <90 mmHg [12]	Measurement of blood pressure (mean of the second and third readings) for the known HT

^a^HT: hypertension.

Because of the multistage stratified random cluster sampling, a 3-stage weighting procedure will be applied to account for (1) the fixed number of villages selected in each OD, (2) the fixed number of households selected in each village, and (3) the number of household members aged 40 and above in each selected household [25,30]. The statistic program Stata 14.2 (StataCorp) [42] will be used to perform the quantitative analyses.

Variables used to generate the cascade of care for T2D and HT are shown in Tables 2 and 3, respectively. The explanatory variables are detailed in Multimedia Appendix 6 [43-47].

The population in the catchment areas is not confined to seek care only in the designated public health facility. To address this, we will also collect patient registry data from the public health facilities in the study setting with regard to patients receiving treatment for triangulating the care model selected.

### Ethics Approval

This study protocol has been approved by the National Ethics Committee for Health Research in Cambodia (reference number 105 NECHR) and by the Institutional Review Board of Institute of Tropical Medicine (Antwerp; reference number 1323/19). The study is also registered as part of the SCUBY protocol at the International Standard Randomised Controlled Trials Number (ISRCTN) registry, number ISRCTN41932064 (first date of publication February 3, 2020).

## Results

Data collection was carried out from mid-July to mid-October 2020. By June 2021, the data cleansing process was finished and cleaned data were properly managed as data sets. As of December 2021, the recruitment process was completed, with 5072 eligible individuals participating in the data collection; however, data analysis is pending. Results are expected to be fully available in mid-2022.

## Discussion

This protocol aims to assess the performance of the 3 dominant care models for T2D and HT through the cascade of care framework, using the population-based survey. This framework will allow us to identify the leakages in the system and the unmet need for care [24]. In addition, we will be able to better understand the diversity in service models across the country by comparing 3 different care models. The design of this study, using large-scale primary data, is unique. The evidence generated from this large-scale survey of more than 5000 households will stimulate policy-relevant analysis that is informative to the existing care for T2D and HT provided by the 3 main care models and act as baseline data for progress monitoring purposes [24]. Identifying gaps in the health system is vital to improve efficiency and effectiveness of its performance.

The strengths of the study are primary data collection, a large sample size, and multiple types of data. This allows us to assess multiple outcomes and to link them with other indicators such as health care utilization, health seeking behavior, morbidity profile, and sociodemographic characteristics. The limitations relate to its complicated set up. The multilevel stratification and the collection of multiple types of (outcome) data make the research design and practical organization difficult. The clustering on more than 2 levels and the different outcomes make it challenging to calculate an ideal sample size following all regulations. We have addressed this by seeking optimal balance between maximizing precision and minimizing costs for feasibility. The purposive selection of ODs based on the mapping of existing care models limits the generalizability of results. However, through randomization within ODs, we will strive for maximum internal validity.

Despite these limitations, this study protocol has a large potential to produce evidence of the performance of different care models for T2D and HT in Cambodia. These insights will help implementers and policy makers to assess scale up and adapt strategies. This is of vital importance owing to the increasing burden of CVDs, T2D, and HT in the country [5,12,13]. As many LMICs struggle with similar burdens of disease and similar structural problems in their health systems, the study protocol and its expected results are also useful for monitoring and scaling up of care for highly prevalent chronic diseases across the globe.

## Appendix

Multimedia Appendix 1 Questionnaire about household information.

Multimedia Appendix 2 Questionnaire about eligible individual information.

Multimedia Appendix 3 Sample list of the eligible households in each village.

Multimedia Appendix 4 Sample record book of anthropometric and biochemical measurements of all the eligible individuals.

Multimedia Appendix 5 Sample record book of HbA_1c_ and Creatinine measurements of known T2D patients or the eligible individual having FBG ≥126 mg/dl.

Multimedia Appendix 6 Identified explanatory variables.

## Abbreviations

CVD: cardiovascular disease
FBG: fasting blood glucose
HbA_1c_: glycated hemoglobin A_1c_
HT: hypertension
LMIC: low- and middle-income country
NCD: noncommunicable disease
OD: operational district
PHC: primary health care
SCUBY: Scale up diabetes and hypertension care for vulnerable people in Cambodia, Slovenia and Belgium
STEPS: STEPwise approach to NCD risk factor surveillance
T2D: type 2 diabetes
WHO PEN: World Health Organization Package of Essential Noncommunicable Disease Interventions

## Data Availability

The data sets generated during or analyzed during this study are available from the corresponding author on reasonable request.
