# Supplementary material for: Generation of Cascades of Care for Diabetes and Hypertension Care Continuum in Cambodia: Protocol for a Population-Based Survey Protocol
Source: JMIR Res Protoc. 2022 Sep 2;11(9):e36747. doi: 10.2196/36747 (PMC9482065; doi:10.2196/36747)
Supplement: Multimedia Appendix 1 [file resprot_v11i9e36747_app1.pdf]

Multimedia Appendix 1. Questionnaire about Household Information

Household Questionnaire

INFORMED CONSENT

INTRO:
Hello, my name is ____and I am from the National Institute of Public Health. Your household has been randomly selected to participate in a study on the scaling up of diabetes and hypertension in Cambodia. This survey is financially supported by the VLIR-UOS project, a Belgian Directorate General for Development grant, through the University of Antwerp. We would like to speak to the household head. The information you give will be kept confidential and no personal details will appear in any record. This interview will take approximately 30 minutes. You do not have to answer any question you don't want to and you can stop the interview at any time. We very much appreciate your participation.

ELIGIBLILTY AND RECRUITMENT CHECK

Q1. Are you a usual member of the household who have stayed in the household the night before the interview or had not been absent for more than 6 months?

[1] Yes
[0] No **(Stop the interview)**

Q2. Are you 18 or above?

[1] Yes
[0] No **(Stop the interview)**

Q3. Are you physically and mentally capable to answer the questions?

[1] Yes
[0] No **(Stop the interview)**

Q4. Are you willing to take part in the study?

[1] Yes
[0] No **(Stop the interview)**

HOUSEHOLD MEMBERS CHARACTERISTICS

A **household** is defined as a person or group of related and unrelated persons who live together in the same dwelling unit(s) or in connected premises, who acknowledge one adult member as the head of the household, and who have common arrangements for cooking and eating meals.

Household members are all usual residents of the household (live under the same roof and share meals) and have stayed in the household the night before the interview or not been absent for more than 6 months.

Q5. How many household members are there in your household? ______ members

		501	502	503	504	505	506	507	508	509
ID in HH	Name	What is the relation of [NAME] with the head of HH?<br><br>*1=Household head<br>2=Wife or Husband<br>3=Son or Daughter<br>4=Son-in-law or Daughter-in-law<br>5=Grandchild<br>6=Parent<br>7=Parent-in-law<br>8=Brother or Sister<br>9=Other relative<br>10=Adopted or Fostered or Stepchild<br>11=Not related*	What is his/her gender?<br><br>*1=Male<br>2=Female*	What is his/her age in complete year?<br><br>*If <1 year, record 0*	What is his/her marital status?<br><br>*1=Married or living together<br>2=Divorced or separated<br>3=Widowed<br>4=Never married and never lived together*	What is his/her current main occupation?<br><br>*1=Household tasks<br>2=Civil servant<br>3=Employee of private company/NGO<br>4=Self-employed farmer<br>5=Large-scale farmer with employees<br>6=Self-employed in small business<br>7=Running a big business with employees<br>8=Casual worker<br>9=Working abroad<br>10=At school (pupil/student)<br>11=Unemployed or not eligible<br>12=Retired*	What is his/her highest educational level?<br><br>*1=No formal education<br>2=Less than primary school<br>3=Primary school<br>4=Secondary school<br>5=High school<br>6=College/University<br>7=Post graduate degree*	In general, how would you describe his/her health status?<br>*1=Good (rarely gets ill)<br>2=Fair (occasionally gets ill)<br>3=Bad (chronically and/or frequently ill)*	Is s/he holding an NSSF card (Any of the 3 types of NSSF card)?<br><br>1=Yes<br>0=No<br><br>(Ask to see the card or refer to the color if possible)	Is s/he listed in Equity Card or Priority Access Card (which entitles him/her to HEF support)?<br><br>1=Yes<br>0=No<br><br>(Ask to see the card and check the name in the Card if possible)
01										
02										
03										
04										
05										
06										
07										
08										
09										
10										

HOUSEHOLD SOCIO-ECONOMIC STATUS

Q6. Does your household have?		
601. electricity	1=Yes	0=No
602. a radio?	1=Yes	0=No
603. a television?	1=Yes	0=No
604. a mobile telephone?	1=Yes	0=No
605. If yes, is it a smartphone?	1=Yes	0=No
606. a refrigerator?	1=Yes	0=No
607. a wardrobe?	1=Yes	0=No
608. a sewing machine or loom?	1=Yes	0=No
609. CD/DVD player?	1=Yes	0=No
610. a generator/battery/solar panel?	1=Yes	0=No

Does any member of your household own/have ?		
611. a watch?	1=Yes	0=No
612. a bicycle or cyclo?	1=Yes	0=No
613. a motorcycle or motor-scooter?	1=Yes	0=No
614. a motorcycle-cart	1=Yes	0=No
615. a oxcart or horsecart?	1=Yes	0=No
616. a car or truck, tractor or van?	1=Yes	0=No
617. a boat with a motor?	1=Yes	0=No
618. a boat without a motor?	1=Yes	0=No
619. any agricultural land?	1=Yes	0=No
620. any livestock, herds, other farm animals, or poultry?	1=Yes	0=No
621. a bank account?	1=Yes	0=No

622	What is the main material of the floor?<br>*Record observation*	1. Earth/sand/clay<br>2. Dung<br>3. Wood planks<br>4. Palm/bamboo<br>5. Parquet or polished wood<br>6. Vinyl or Asphalt strips<br>7. Ceramic tiles<br>8. Cement tiles<br>9. Cement<br>10. Floating house<br>11. Other (specify)
623	What is the main material of the roof?<br>*Record observation*	1. Bamboo/thatch/palm leaf<br>2. Rustic mat<br>3. Wood planks<br>4. Cardboard<br>5. Plastic sheet<br>6. Metal<br>7. Wood<br>8. Calamine/cement fiber<br>9. Ceramic tiles<br>10. Clay tiles<br>11. Cement<br>12. Other (specify)
624	What is the main material of the exterior walls?<br>*Record observation*	1. Palm/bamboo/thach<br>2. Dirt<br>3. Bamboo with mud<br>4. Straw with mud<br>5. Stone with mud<br>6. Uncovered adobe

		7. Plywood<br>8. Cardboard<br>9. Reused wood<br>10. Metal<br>11. Cement<br>12. Stone with lime/cement<br>13. Bricks<br>14. Cement blocks<br>15. Covered adobe<br>16. Wood planks/shingles<br>17. Other (specify)
625	How many rooms in this household are used for sleeping?	Rooms:…………………
626	What is the main source of drinking water during the wet season for members of your household?	1. Piped into dwelling<br>2. Piped to yard/plot<br>3. Public tap/standpipe<br>4. Tube well or borehole<br>5. Protected well<br>6. Unprotected well<br>7. Protected spring<br>8. Unprotected spring<br>9. Rainwater<br>10. Tanker truck<br>11. Cart with small tank<br>12. Surface water (river/dam/lake/pond/stream/canal/irrigation channel<br>13. Bottled water<br>14. Other (specify)
627	What is the main source of drinking water during the dry season for members of your household?	1. Piped into dwelling<br>2. Piped to yard/plot<br>3. Public tap/standpipe<br>4. Tube well or borehole<br>5. Protected well<br>6. Unprotected well<br>7. Protected spring<br>8. Unprotected spring<br>9. Rainwater<br>10. Tanker truck<br>11. Cart with small tank<br>12. Surface water (river/dam/lake/pond/stream/canal/irrigation channel<br>13. Bottled water<br>14. Other (specify)
628	Do you do anything to the water to make it safer to drink?	1. Yes, always<br>2. Yes, sometime<br>3. No<br>4. Don’t know
629	What do you usually do to make the water safer to drink?<br>*Record all mentioned.*	1. Boil<br>2. Add bleach/chlorine<br>3. Strain through a cloth<br>4. Use water filter (ceramic/sand/composite/etc.)<br>5. Solar disinfection<br>6. Let it stand and settle<br>7. Other (specify)

630	What kind of toilet facility do members of your household usually use?	1. Flush to piped sewer system (not shared with other households)<br>2. Flush to septic tank (not shared with other households)<br>3. No facility/bush/field<br>4. Other type of toilet (specify)

GENERAL MORBIDITY, ACCESS TO HEALTH CARE, RELATED HEALTH EXPENDITURE AND COPING STRATEGIES

Q7. *701-In the past month, were you or any member of the household ill (including those with chronic diseases and abortion) or injured?*

[1]=Yes
[0]=No (***Skip to Q8***)
[99]=Don't Know (***Skip to Q8***)

702-If yes, how many persons, including you? __ __ (99 if Don't Know)
703-If more than one person, ask for [NAME] of the last one and write down his/her ID in HH here: ___/___

Now let me ask you about health care seeking and related expenditure of [NAME],
704-Did [NAME] seek care for his/her reported illness/injury?

[1]=Yes
[0]=No (***Skip to 711)***
[99]=Don't Know (***Skip to 711***)

705-If Yes, how many times? _____/_____
706-Where did [NAME] seek care for the **FIRST TIME**?

[01] = National hospital (PP)
[02] = Provincial hospital (RH)
[03] = District hospital (RH)
[04] = Health centre
[05] = Health post
[06] = Provincial rehabilitation centre (PRC) or Community-based rehabilitation (CBR)
[07] = Other public; specify: ___________
[08] = Private hospital
[09] = Private clinic
[10] = Private pharmacy
[11] = Home/Office of trained health worker/nurse
[12] = Visit of trained health worker/nurse
[13] = Other private medical; specify: ___________
[14] = Shop selling drugs/market
[15] = Kru Khmer/ Magician
[16] = Monk/religious leader
[17] = Traditional birth attendant
[18] = Oversee medical service
[19] = Other; specify: ___________

707-Where did [NAME] seek care for the **LAST TIME**? ***(If answer to Q405 was more than once)***

[01] = National hospital (PP)
[02] = Provincial hospital (RH)
[03] = District hospital (RH)
[04] = Health centre
[05] = Health post
[06] = Provincial rehabilitation centre (PRC) or Community-based rehabilitation (CBR)
[07] = Other public; specify: ____________
[08] = Private hospital
[09] = Private clinic
[10] = Private pharmacy
[11] = Home/Office of trained health worker/nurse
[12] = Visit of trained health worker/nurse
[13] = Other private medical; specify: ____________
[14] = Shop selling drugs/market
[15] = Kru Khmer/ Magician
[16] = Monk/religious leader
[17] = Traditional birth attendant
[18] = Oversee medical service
[19] = Other; specify: ___________

708-How much in total did [NAME] or your household spend for his/her care and treatment, including payment to providers and transportation to go for the care & treatment? _______________________ Riels

709-Did [NAME] or your household receive any financial support, including payment for user fees (often in the form of free care and transportation) from HEF/NSSF for his/her care and treatment?

[1]=Yes
[0]=No
[99]=Don’t know

710-A part from HEF/NSSF support, if any, what were the 3 major sources of financing the expenditure? Put no 1, 2, 3 according to the amount spent and put 0 for No

Household income	[___]
Savings	[___]
Borrowing or using loan	[___]
Selling assets	[___]
Selling household production in advance	[___]
Other	[___]; Specify: ______________

711-If No, any self-medication at home?

[1]=Yes
[0]=No
[99]=Don't Know

Q8. *801-In the past 12 months (including last month), were you or any member of the household hospitalized (one overnight stay in hospital or any health care facility, EXCEPT FOR DELIVERY)?*

[1]=Yes
[0]=No (*Skip to Q9*)
[99]=Don't Know (*Skip to Q9*)

802-If yes**, how many persons,** including you? _____ (99 if Don't Know)
803-If more than one person, ask for [NAME] of the last one and write down his/her ID in HH here: ____/____
804-Where was [NAME] hospitalized for the **LAST TIME**?

[01] = National hospital (PP)
[02] = Provincial hospital (RH)
[03] = District hospital (RH)
[04] = Health centre
[05] = Health post
[06] = Provincial rehabilitation centre (PRC) or Community-based rehabilitation (CBR)
[07] = Other public; specify: ___________
[08] = Private hospital
[09] = Private clinic
[10] = Private pharmacy
[11] = Home/Office of trained health worker/nurse
[12] = Visit of trained health worker/nurse
[13] = Other private medical; specify: ____________
[14] = Shop selling drugs/market
[15] = Kru Khmer/ Magician
[16] = Monk/religious leader
[17] = Traditional birth attendant
[18] = Oversee medical service
[19] = Other; specify: __________

805-How much in total did [NAME] or your household spend for the hospitalization, including payment to providers, transportation to go to the hospital and back home and food during the stay in the hospital? __________ Riels

806-Did [NAME] or your household received any financial support, including payment for user fees (often in the form of free care) and transportation) from HEF/NSSF for his/her care and treatment?

[1]=Yes
[0]=No
[99]=Don’t know

807-A part from HEF/NSSF support, if any, what were the 3 major sources of financing the expenditure? Put no 1, 2, 3 according to the amount spent and put 0 for No

Household income	[___]
Savings	[___]
Borrowing or using loan	[___]
Selling assets	[___]
Selling household production in advance	[___]
Other	[___] Specify: ______________

Q9. *901 -Has anybody in this household used any of the following services in the past 12 months?*

a. Family planning services

  [1] =Yes

  [0] =No

  [99]=Don't know

b. Antenatal care

  [1]=Yes

  [0]=No

  [99]=Don't know

c. Normal delivery and associated services

  [1]=Yes

  [0]=No

  [99]=Don't know

d. Postnatal care

  [1]=Yes

  [0]=No

  [99]=Don't know

e. Vaccination services for women and children

  [1]=Yes

  [0]=No

  [99]=Don't know

If No or Don't Know for all the services, STOP the questionnaire here.

902-If yes, how many persons, including you? _______ (99 if Don't Know)

903-If more than one person, ask for [NAME] of the last one and write down his/her ID in HH here: ____/____

904-Where did [NAME] seek the indicated preventive care for the **LAST TIME**?

[01] = National hospital (PP)
[02] = Provincial hospital (RH)
[03] = District hospital (RH)
[04] = Health centre
[05] = Health post
[06] = Provincial rehabilitation centre (PRC) or Community-based rehabilitation (CBR)
[07] = Other public; specify: ___________
[08] = Private hospital
[09] = Private clinic
[10] = Private pharmacy
[11] = Home/Office of trained health worker/nurse
[12] = Visit of trained health worker/nurse
[13] = Other private medical; specify: ____________

[14] = Shop selling drugs/market
[15] = Kru Khmer/ Magician
[16] = Monk/religious leader
[17] = Traditional birth attendant
[18] = Oversee medical service
[19] = Other; specify: __________

905-How much in total did [NAME] or your household spend for the preventive care, including payment to providers and transportation to go for the care & treatment? ____________ Riels

906-Did [NAME] or your household received any financial support, including payment for user fees (often in the form of free care and transportation) from HEF/NSSF for his/her care and treatment?
[1]=Yes
[0]=No
[99]=Don't know

907-A part from HEF/NSSF support, if any, what were the 3 major sources of financing the expenditure? Put no 1, 2, 3 according to the amount spent and put 0 for No

Household income	[___]
Savings	[___]
Borrowing or using loan	[___]
Selling assets	[___]
Selling household production in advance	[___]
Other	[___] Specify: _____________

GPS of household location
Latitude: ________
Longitude: ________

[End of questionnaire]
