# Supplementary material for: Generation of Cascades of Care for Diabetes and Hypertension Care Continuum in Cambodia: Protocol for a Population-Based Survey Protocol
Source: JMIR Res Protoc. 2022 Sep 2;11(9):e36747. doi: 10.2196/36747 (PMC9482065; doi:10.2196/36747)
Supplement: Multimedia Appendix 2 [file resprot_v11i9e36747_app2.pdf]

Multimedia Appendix 2. Questionnaire about Eligible Individual Information

Individual Questionnaire

INFORMED CONSENT

INTRO:
Hello, my name is ____and I am from the National Institute of Public Health. You has been randomly selected to participate in this study [on the scaling up of diabetes and hypertension in Cambodia] based on information from your household. The information you give will be kept confidential and no personal details will appear in any record. This interview will take approximately 60 minutes. You do not have to answer any question you don't want to and you can stop the interview at any time. We will also measure your blood pressure, weight and height, waist and hip circumferences and your fasting blood glucose (FBG) [and HbA1c and Creatinine level for known diabetes and those having FBG ≥ 126 mg/dl]. We very much appreciate your participation and information.

ELIGIBILITY AND RECRUITMENT CHECK

Q1. Are you a usual member of the household who have stayed in the household the night before the interview or had not been absent for more than 6 months?
[1] Yes
[0] No **(Stop the interview)**

Q2. Are you 40 or above?
[1] Yes
[0] No **(Stop the interview)**

Q3. Are you physically and mentally capable to answer the questions?
[1] Yes
[0] No **(Stop the interview)**

Q4. Are you willing to take part in the study?
[1] Yes
[0] No **(Stop the interview)**

SECTION 1: SOCIO-DEMOGRAPHIC INFORMATION

**Q.N**	**Description & questions**	**Response**
Q5	How old are you?<br>*-Record in years as stated by the participant*<br>*-Record 99 if don't know*	____________Years
Q6	Sex of participant	1 = Male

	Record sex of the participant as observed	2 = Female
Q7	What is your marital status?<br><br>*-Record 88 if refuse to answer*	1=Married or living together<br>2=Divorced or separated<br>3=Widowed<br>4=Never married and never lived together
Q8	What is your highest educational level?<br><br>*Record 99 if don't know*	1=No formal schooling<br>2=Less than primary school<br>3=Primary school<br>4=Secondary school<br>5=High school<br>6=College/University<br>7=Post graduate degree
Q9	What is your ethnic group?	1 = Khmer<br>2 = Vietnamese<br>3 = Chinese<br>4 = Cham (Muslim)<br>5 = Other
Q10	Which of the following best describes your main work status within the past 12 months?<br><br>*Record 88 if refuse to answer*	1=Household tasks<br>2=Civil servant<br>3=Employee of private company/NGO<br>4=Self-employed farmer<br>5=Large-scale farmer with employees<br>6=Self-employed in small business<br>7=Running a big business with employees<br>8=Casual worker<br>9=Working abroad<br>10=At school (pupil/student)<br>11=Unemployed or not eligible<br>12=Retired
Q11	Taking the past year, can you give an estimate of your annual income if I read some options to you?<br><br>*Record 88 if refuse to answer*	1 = no earnings<br>2 = less than or 250 USD<br>3 = more than 251- 1500 USD<br>4 = more than 1501 – 3500 USD<br>5 = more than 3501 USD

SECTION 2: HEALTH STATUS AND QUALITY OF LIFE

**Q.N**	**Description and Questions**	**Response**
Q12	At this point of time in your life, how would you describe:	1 = Excellent<br>2 = Very good<br>3 = Good

	Q 12.1. Your home situation [____]<br>Q 12.2. Your family relationships [____]<br>Q 12.3. Your finances [____]<br>Q 12.4. Your work situation [____]<br><br>*-Record 99 if don't know and 88 if refuse*	4 = Fair<br>5 = Poor
Q13	How good or bad is your health today?<br><br>*-The scale is numbered from 0 to 100.*<br>*-100 means the best health you can imagine.*<br>*-0 means the worst health you can imagine.*<br>*-Please mark an X on the scale to indicate how your health is today.*<br><br>*-Record 99 if don't know and 88 if refuse*	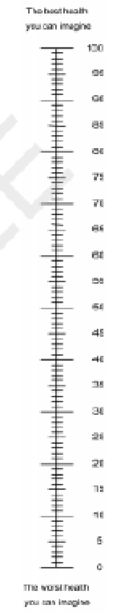
Q14	Have you ever been told by a doctor or other health worker that you have hypertension?<br><br>*-Record 99 if don't know/unsure*	0 = No<br>1 = Yes
Q15	Have you ever been told by a doctor or other health worker that you have diabetes?<br><br>*-Record 99 if don't know/unsure*	0 = No<br>1 = Yes
Q16	Have you ever been told by a doctor or other health worker that you have heart problems?<br><br>*-Record 99 if don't know/unsure*	0 = No<br>1 = Yes
Q17	Have you ever been told by a doctor or other health worker that you have symptoms suggestive of a stroke?<br><br>*-Record 99 if don't know/unsure*	0 = No<br>1 = Yes

Q18	Have you ever been told by a doctor or other health worker that you have chronic kidney disease?<br><br>*-Record 99 if don't know/unsure*	0 = No<br>1 = Yes
Q19	How many natural teeth do you have?<br><br>*-Record 99 if don't know*	0 = None<br>1 = 1-9 teeth<br>2 = 10-19 teeth<br>3 = 20 teeth or more
Q20	During the past 12 months, did your teeth or mouth cause any pain or comfort?<br><br>*-Record 99 if don't know*	0 = No<br>1 = Yes
Q21	Over the last 2 weeks, how often have you been bothered by any of the following problems?<br><br>Q 21.1. Little interest or pleasure in doing things [____]<br>Q 21.2. Feeling down, depressed, or hopeless. [____]<br>Q 21.3. Trouble falling or staying asleep, or sleeping too much. [____]<br>Q 21.4. Feeling tired or having little energy. [____]<br>Q 21.5. Poor appetite or overeating [____]<br>Q 21.6. Feeling bad about yourself – or that you are a failure or make yourself or down your family [____]<br>Q 21.7. Trouble concentrating on things, such as reading the newspaper or watching television [____]<br>Q 21.8. Moving or speaking so slowly that other people could have noticed? Or the oppositebeing so fidgety or restless that you have been moving around a lot more than usual [____]<br>Q 21.9. Thoughts that you would be better off dead or of hurting yourself in some ways [____]	0 = Not at all<br>1 = Several days<br>2 = More than half the days<br>3 = Nearly everyday

Q22	We would like to confirm that you have:<br><br>*-This question is for categorizing respondents for the following sections and it is also important to ask respondents to confirm their main conditions in this survey.*<br><br>*-IF the answer is "0", go to Section 3*<br>*-IF the answer is "1", go to Section 3a*<br>*-IF the answer is "2", go to Section 3b*<br>*-IF the answer is "3", go to Section 3c*	0 = Neither hypertension nor diabetes<br><br>1 = Only hypertension<br><br>2 = Only diabetes<br><br>3 = Both diabetes and hypertension

SECTION 3: HEALTH CARE UTILIZATION

Q.N	Description and Questions	Response
Q23	Have you sought medical treatment or advice as an outpatient from anyone in the past 3 months?<br><br>*-If No, go to Question 40.*	0 = No<br>1 = Yes
Q24	Where did you seek medical advice or treatment for illness in the past 3 months?<br><br>*-More than one answer can be selected.*<br>*-Data collectors can use probes to help respondents determine the types of health facilities in the Response Column.*<br>*-Record 99 if don't know and 88 if refuse*	1= National hospital (PP)<br>2= Provincial hospital (RH)<br>3= District hospital (RH)<br>4= Health centre<br>5= Health post<br>6= Provincial rehabilitation centre (PRC) or Community-based rehabilitation (CBR)<br>7= Other public; specify:<br>8= Private hospital<br>9= Private clinic<br>10= Private pharmacy<br>11= Home/Office of trained health worker/nurse<br>12= Visit of trained health worker/nurse<br>13= Other private medical<br>14= Shop selling drugs/market<br>15= Kru Khmer/ Magician<br>16= Monk/religious leader<br>17= Traditional birth attendant<br>18= Oversee medical service<br>19= Other; specify

From Q25-Q39, it is a set of questions that are asked following choices selected in Q24. If 2 or 3 choices were selected in Q24, Q25-Q39 would appear 2 or 3 times, accordingly.		
Q25	How many times did you visit the selected place**(s)** in Q24 in the past three months?	_______ times
Q26	How much in total was spent on the treatment at the selected place(s) in Q24?<br><br>*-Record 99 if don't know and 88 if refuse*	0 = free/no cost<br>1 = in kind<br>2 = ________Riels
Q27	How did you pay for the treatment cost at the selected place(s) in Q24?<br><br>*-Record 99 if don't know and 88 if refuse*	1= Health Equity Fund<br>2= Voucher<br>3= Fee Exemption<br>4= NGO<br>5= National Social Security Fund<br>6= Community-Based Health Insurance<br>7= Health Insurance through Employer<br>8= Other Privately Purchased Commercial Health Insurance<br>9= Wage/income<br>10= Loan/ Ton Tin<br>11= Sale of Assets<br>12= Gift from Relative<br>13= Savings<br>14= Other
Q28	How much in total was spent on transport to go to and return from the selected place(s) in Q24?<br><br>*-Record 99 if don't know and 88 if refuse*	0 = free/no cost<br>1 = in kind<br>2 = ________Riels
Q29	On average how many hours do you spend to get treatment/advices from the selected place(s) in Q24?<br><br>*-Record 99 if don't know and 88 if refuse*	________Hours

Q30	How satisfied are you with the effect of your {treatment/care} at the selected place(s) in Q24?<br><br>*-Record 99 if don't know/unsure*	0 = Very satisfied<br>1 = Satisfied<br>2 = Neither satisfied nor dissatisfied<br>3 = Dissatisfied<br>4 = Very dissatisfied
Q31	How satisfied are you with the explanations the {doctor/other health professional} has given you about the results of your {treatment/care} at the selected place(s) in Q24?<br><br>*-Record 99 if don't know/unsure*	0 = Very satisfied<br>1 = Satisfied<br>2 = Neither satisfied nor dissatisfied<br>3 = Dissatisfied<br>4 = Very dissatisfied
Q32	The {doctor/other health professional} at the selected place(s) in Q24 was very careful to check everything when examining you.<br><br>*-Record 99 if don't know/unsure*	0 = Strongly agree<br>1 = Agree<br>2 = Not sure<br>3 = Disagree<br>4 = Strongly disagree
Q33	At the selected place(s) in Q24, how satisfied were you with the choices you had in decisions affecting your health care?<br><br>*-Record 99 if don't know/unsure*	0 = Very satisfied<br>1 = Satisfied<br>2 = Neither s<br>atisfied nor dissatisfied<br>3 = Dissatisfied<br>4 = Very dissatisfied
Q34	How much of the time did you feel respected by the {doctor/other health professional} at the selected place(s) in Q24?<br><br>*-Record 99 if don't know/unsure*	0 = All of the time<br>1 = Most of the time<br>2 = About half the time<br>3 = Some of the time<br>4 = None of the time
Q35	At the selected place(s) in Q24, the time you had with the {doctor/other health professional} was too short.<br><br>*-Record 99 if don't know/unsure*	0 = Strongly agree<br>1 = Agree<br>2 = Not sure<br>3 = Disagree<br>4 = Strongly disagree

Q36	Are you satisfied with the care you received in the selected place(s) in Q24?<br><br>*-Record 99 if don't know/unsure*	0 = Very satisfied<br>1 = Satisfied<br>2 = Neither s<br>atisfied nor dissatisfied<br>3 = Dissatisfied<br>4 = Very dissatisfied
Q37	Did you get your blood pressure measured at the selected place(s) in Q24?<br><br>*-Record 99 if don't know/unsure*	0 = No<br>1 = Yes
Q38	Did you get your blood glucose tested at the selected place(s) in Q24?<br><br>*-Record 99 if don't know/unsure*	0 = No<br>1 = Yes
Q39	Would you recommend the selected place(s) in Q24 to others?<br><br>*-Record 99 if don't know/unsure*	1 = Not recommended<br>2 = Recommend with reservations<br>3 = Recommend<br>4 = Highly recommend
Q40	Have you ever had your blood glucose tested in the last three years?<br><br>*-Record 99 if don't know/unsure*	0 = No<br>1 = Yes
Q41	Have you ever had your blood pressure measured in the last three years?<br><br>*-Record 99 if don't know/unsure*	0 = No<br>1 = Yes

SECTION 3a: HEALTH CARE UTILIZATION FOR HYPERTENSION

Q.N	Description and Questions	Response
Q42a	How long have you lived with hypertension?<br><br>*-Record 99 if don't know/unsure and 88 if refuse*<br>*-Less than a year is rounded up to one year*<br>*-Standard rounded up formula is applied.*	________Years
Q43a	Where were you first diagnosed as having hypertension?<br><br>*-Record 99 if don't know and 88 if refuse*	1= National hospital (PP)<br>2= Provincial hospital (RH)<br>3= District hospital (RH)<br>4= Health centre<br>5= Health post<br>6= Provincial rehabilitation centre (PRC) or Community-based rehabilitation (CBR)<br>7= Other public<br>8= Private hospital<br>9= Private clinic<br>10= Private pharmacy<br>11= Home/Office of trained health worker/nurse<br>12= Visit of trained health worker/nurse<br>13= Other private medical<br>14= Shop selling drugs/market<br>15= Kru Khmer/ Magician<br>16= Monk/religious leader<br>17= Traditional birth attendant<br>18= Oversee medical service<br>19= MoPoTsyo
Q44a	Where did you first seek advice or treatment for hypertension after being diagnosed?<br><br>*-Record 99 if don't know and 88 if refuse*	1= National hospital (PP)<br>2= Provincial hospital (RH)<br>3= District hospital (RH)<br>4= Health centre<br>5= Health post<br>6= Provincial rehabilitation centre (PRC) or Community-based rehabilitation (CBR)<br>7= Other public<br>8= Private hospital<br>9= Private clinic<br>10= Private pharmacy<br>11= Home/Office of trained health worker/nurse

		12= Visit of trained health worker/nurse<br>13= Other private medical<br>14= Shop selling drugs/market<br>15= Kru Khmer/ Magician<br>16= Monk/religious leader<br>17= Traditional birth attendant<br>18= Oversee medical service<br>19= MoPoTsyo<br>20= Other; specify
Q45a	Did you go to other places for follow up treatment/care for your hypertensive conditions?<br><br>*-Record 88 if refuse*<br>*-If NO, please skip Q46a*	0 = No<br>1 = Yes
Q46a	If yes to Q46a, where else did you go to get follow up treatment/care for your hypertensive conditions?	1= National hospital (PP)<br>2= Provincial hospital (RH)<br>3= District hospital (RH)<br>4= Health centre<br>5= Health post<br>6= Provincial rehabilitation centre (PRC) or Community-based rehabilitation (CBR)<br>7= Other public<br>8= Private hospital<br>9= Private clinic<br>10= Private pharmacy<br>11= Home/Office of trained health worker/nurse<br>12= Visit of trained health worker/nurse<br>13= Other private medical<br>14= Shop selling drugs/market<br>15= Kru Khmer/ Magician<br>16= Monk/religious leader<br>17= Traditional birth attendant<br>18= Oversee medical service<br>19= MoPoTsyo<br>20= Other; specify:

Q47a	Did you get treatment/care for your hypertensive conditions in the past **12 months?**<br><br>*-Record 88 if refuse*<br>*-If NO, please skip Q48a-63a*	0 = No<br>1 = Yes
Q48a	Are you currently receiving any of the following treatment/advices for your hypertensive conditions prescribed by a doctor or other health care worker?<br><br>Q 48.1a. Drugs (medication) that you have taken in the past two weeks [____]<br>Q 48.2a. Advice to reduce salt intake [___]<br>Q 48.3a. Advice or treatment to lose weight [____]<br>Q 48.4a. Advice or treatment to stop smoking [____]<br>Q 48.5a. Advice to start or do more physical exercise [____]<br>Q 48.6a. Advice to stop drinking alcohol [____]<br><br>*-Record 99 if don't know and 88 if refuse*	0 = No 1= Yes
Q49a	Have you had your blood cholesterol measured in the past 12 months?<br><br>*-Record 99 if don't know and 88 if refuse*	0 = No 1= Yes
Q50a	Where did you seek medical advice or treatment for your hypertensive condition in **the past 3 months**?<br><br>***-More than one answer can be selected****.*<br>*-Data collectors can use probes to help respondents determine the types of health facilities in the Response Column.*<br>*-Record 99 if don't know and 88 if refuse*	1= National hospital (PP)<br>2= Provincial hospital (RH)<br>3= District hospital (RH)<br>4= Health centre<br>5= Health post<br>6= Provincial rehabilitation centre (PRC) or Community-based rehabilitation (CBR)<br>7= Other public<br>8= Private hospital<br>9= Private clinic<br>10= Private pharmacy<br>11= Home/Office of trained health worker/nurse<br>12= Visit of trained health worker/nurse<br>13= Other private medical

		14= Shop selling drugs/market<br>15= Kru Khmer/ Magician<br>16= Monk/religious leader<br>17= Traditional birth attendant<br>18= Oversee medical service<br>19= MoPoTsyo
From Q51a-Q63a, it is a set of questions that are asked following choices selected in Q51a. If 2 or 3 choices were selected in Q50a, Q51a-Q63a would appear 2 or 3 times, accordingly.		
Q51a	How many times did you visit the selected place**(s)** in Q50a in the past three months?	_______ times
Q52a	How much in total was spent on the treatment at the selected place(s) in Q50a?<br><br>*-Record 99 if don't know and 88 if refuse*	0 = free/no cost<br>1 = in kind<br>2 = ________Riels
Q53a	How did you pay for the treatment cost at the selected place(s) in Q50a?<br><br>*-Record 99 if don't know and 88 if refuse*	1= Health Equity Fund<br>2= Voucher<br>3= Fee Exemption<br>4= NGO<br>5= National Social Security Fund<br>6= Community-Based Health Insurance<br>7= Health Insurance through Employer<br>8= Other Privately Purchased Commercial Health Insurance<br>9= Wage/income<br>10= Loan/ Ton Tin<br>11= Sale of Assets<br>12= Gift from Relative<br>13= Savings<br>14= Other

Q54a	How much in total was spent on transport to go to and return from the selected place(s) in Q50a?<br><br>*-Record 99 if don't know and 88 if refuse*	0 = free/no cost<br>1 = in kind<br>2 = ________Riels
Q55a	On average how many hours do you spend to get treatment/advices from the selected place(s) in Q50a?<br><br>*-Record 99 if don't know and 88 if refuse*	________Hours
Q56a	How satisfied are you with the effect of your {treatment/care} at the selected place(s) in Q50a?<br><br>*-Record 99 if don't know/unsure*	0 = Very satisfied<br>1 = Satisfied<br>2 = Neither satisfied nor dissatisfied<br>3 = Dissatisfied<br>4 = Very dissatisfied
Q57a	How satisfied are you with the explanations the {doctor/other health professional} has given you about the results of your {treatment/care} at the selected place(s) in Q50a?<br><br>*-Record 99 if don't know/unsure*	0 = Very satisfied<br>1 = Satisfied<br>2 = Neither satisfied nor dissatisfied<br>3 = Dissatisfied<br>4 = Very d<br>issatisfied
Q58a	The {doctor/other health professional} at the selected place(s) in Q50a was very careful to check everything when examining you.<br><br>*-Record 99 if don't know/unsure*	0 = Strongly agree<br>1 = Agree<br>2 = Not sure<br>3 = Disagree<br>4 = Strongly disagree
Q59a	At the selected place(s) in Q50a, how satisfied were you with the choices you had in decisions affecting your health care?<br><br>*-Record 99 if don't know/unsure*	0 = Very satisfied<br>1 = Satisfied<br>2 = Neither s<br>atisfied nor dissatisfied<br>3 = Dissatisfied<br>4 = Very dissatisfied

Q60a	How much of the time did you feel respected by the {doctor/other health professional} at the selected place(s) in Q50a?<br><br>*-Record 99 if don't know/unsure*	0 = All of the time<br>1 = Most of the time<br>2 = About half the time<br>3 = Some of the time<br>4 = None of the time
Q61a	At the selected place(s) in Q50a, the time you had with the {doctor/other health professional} was too short.<br><br>*-Record 99 if don't know/unsure*	0 = Strongly agree<br>1 = Agree<br>2 = Not sure<br>3 = Disagree<br>4 = Strongly disagree
Q62a	Are you satisfied with the care you received in the selected place(s) in Q50a?<br><br>*-Record 99 if don't know/unsure*	0 = Very satisfied<br>1 = Satisfied<br>2 = Neither s<br>atisfied nor dissatisfied<br>3 = Dissatisfied<br>4 = Very dissatisfied
Q63a	Would you recommend the selected place(s) in Q50a to others?<br><br>*-Record 99 if don't know/unsure*	1 = Not recommend<br>2 = Recommend with reservations<br>3 = Recommend<br>4 = Highly recommend
Q64a	Have you ever been told by a doctor that you have eyes problems?<br><br>*-Record 99 if don't know/unsure*	0 = No<br>1 = Yes
Q65a	Have you ever been told by a doctor that you have kidney problems?<br><br>*-Record 99 if don't know/unsure*	0 = No<br>1 = Yes

Q66a	Have you ever been told by a doctor that you have lost the sensation of your peripheral membrane, such as foot, hand, arm?<br><br>*-Record 99 if don't know/unsure*	0 = No<br>1 = Yes
Q67a	In **the past 3 months**, besides seeking medical advice or treatment for your hypertensive condition, have you sought medical treatment or advice for other illnesses or conditions?<br><br>If No, go to Section 4.<br>If Yes, go to Q24-Q39	0 = No<br>1 = Yes

SECTION 3b: HEALTH CARE UTILIZATION FOR DIABETES

**Q.N**	**Description and Questions**	**Response**
Q42b	How long have you lived with diabetes?<br><br>*-Record 99 if don't know/unsure and 88 if refuse*<br>*-Less than a year is rounded up to one year*<br>*-Standard rounded up formula is applied.*	________Years
Q43b	Where were you first diagnosed as having diabetes?<br><br>*-Record 99 if don't know and 88 if refuse*	1= National hospital (PP)<br>2= Provincial hospital (RH)<br>3= District hospital (RH)<br>4= Health centre<br>5= Health post<br>6= Provincial rehabilitation centre (PRC) or Community-based rehabilitation (CBR)<br>7= Other public<br>8= Private hospital<br>9= Private clinic<br>10= Private pharmacy<br>11= Home/Office of trained health worker/nurse<br>12= Visit of trained health worker/nurse<br>13= Other private medical<br>14= Shop selling drugs/market<br>15= Kru Khmer/ Magician<br>16= Monk/religious leader<br>17= Traditional birth attendant<br>18= Oversee medical service

		19= MoPoTsyo<br>20= Other; specify:
Q44b	Where did you first seek advice or treatment for diabetes after being diagnosed?<br><br>*-Record 99 if don't know and 88 if refuse*	1= National hospital (PP)<br>2= Provincial hospital (RH)<br>3= District hospital (RH)<br>4= Health centre<br>5= Health post<br>6= Provincial rehabilitation centre (PRC) or Community-based rehabilitation (CBR)<br>7= Other public<br>8= Private hospital<br>9= Private clinic<br>10= Private pharmacy<br>11= Home/Office of trained health worker/nurse<br>12= Visit of trained health worker/nurse<br>13= Other private medical<br>14= Shop selling drugs/market<br>15= Kru Khmer/ Magician<br>16= Monk/religious leader<br>17= Traditional birth attendant<br>18= Oversee medical service<br>19= MoPoTsyo<br>20= Other; specify:
Q45b	Did you go to other places for follow up treatment/care for your diabetes conditions?<br><br>*-Record 88 if refuse*<br>*-If NO, please skip Q46b*	0 = No<br>1 = Yes
Q46b	If yes to Q45b, where else did you go to get follow up treatment/care for your diabetes conditions?	1= National hospital (PP)<br>2= Provincial hospital (RH)<br>3= District hospital (RH)<br>4= Health centre<br>5= Health post<br>6= Provincial rehabilitation centre (PRC) or Community-based rehabilitation (CBR)<br>7= Other public<br>8= Private hospital<br>9= Private clinic<br>10= Private pharmacy<br>11= Home/Office of trained health worker/nurse

		12= Visit of trained health worker/nurse<br>13= Other private medical<br>14= Shop selling drugs/market<br>15= Kru Khmer/ Magician<br>16= Monk/religious leader<br>17= Traditional birth attendant<br>18= Oversee medical service<br>19= MoPoTsyo<br>20= Other; specify:
Q47b	Did you get treatment/care for your diabetes conditions in the past 12 months?<br><br>*-Record 88 if refuse*<br>*-If NO, please skip Q48b-64b*	0 = No<br>1 = Yes
Q48b	Are you currently receiving any of the following treatment/advices for your diabetes conditions prescribed by a doctor or other health care worker?<br><br>Q. 48b.1. Insulin [____]<br>Q. 48b.2. Drugs (medication) that you have taken in the past two weeks [____]<br>Q. 48b.3. Special prescribed diet [____]<br>Q. 48b.4. Advice or treatment to lose weight [____]<br>Q. 48b.5. Advice or treatment to stop smoking<br>Q. 48b.6. Advice to start or do more physical exercise<br>Q. 48b.7.Advice to stop drinking alcohol [____]	0 = No<br>1= Yes
Q49b	Have you had your blood glucose measured in the past 12 months?<br><br>*-Record 99 if don't know and 88 if refuse*	0 = No<br>1= Yes
Q50b	Have you had your HbA1c tested in the past 12 months?<br><br>*-Record 99 if don't know and 88 if refuse*	0 = No<br>1= Yes

Q51b	Where did you seek medical advice or treatment for illness in the **past 3 months**?<br><br>***-More than one answer can be selected.***<br>*-Data collectors can use probes to help respondents determine the types of health facilities in the Response Column.*<br>*-Record 99 if don't know and 88 if refuse*	1= National hospital (PP)<br>2= Provincial hospital (RH)<br>3= District hospital (RH)<br>4= Health centre<br>5= Health post<br>6= Provincial rehabilitation centre (PRC) or Community-based rehabilitation (CBR)<br>7= Other public<br>8= Private hospital<br>9= Private clinic<br>10= Private pharmacy<br>11= Home/Office of trained health worker/nurse<br>12= Visit of trained health worker/nurse<br>13= Other private medical<br>14= Shop selling drugs/market<br>15= Kru Khmer/ Magician<br>16= Monk/religious leader<br>17= Traditional birth attendant<br>18= Oversee medical service<br>19= MoPoTsyo<br>20= Other; specify:
From Q52b-Q64b, it is a set of questions that are asked following choices selected in Q51b. If 2 or 3 choices were selected in Q51b, Q52b-Q64b would appear 2 or 3 times, accordingly.		
Q52b	How many times did you visit the selected place**(s)** in Q51b in the past three months?	_______ times
Q53b	How much in total was spent on the treatment at the selected place(s) in Q51b?<br><br>*-Record 99 if don't know and 88 if refuse*	0 = free/no cost<br>1 = in kind<br>2 = ________Riels OR<br>________ USD

Q54b	How did you pay for the treatment cost at the selected place(s) in Q51b?<br><br>*-Record 99 if don't know and 88 if refuse*	1= Health Equity Fund<br>2= Voucher<br>3= Fee Exemption<br>4= NGO<br>5= National Social Security Fund<br>6= Community-Based Health Insurance<br>7= Health Insurance through Employer<br>8= Other Privately Purchased Commercial Health Insurance<br>9= Wage/income<br>10= Loan/ Ton Tin<br>11= Sale of Assets<br>12= Gift from Relative<br>13= Savings<br>14= Other
Q55b	How much in total was spent on transport to go to and return from the selected place(s) in Q51b?<br><br>*-Record 99 if don't know and 88 if refuse*	0 = free/no cost<br>1 = in kind<br>2 = ________Riels OR<br>________ USD
Q56b	On average how many hours do you spend to get treatment/advices from the selected place(s) in Q51b?<br><br>*-Record 99 if don't know and 88 if refuse*	________Hours
Q57b	How satisfied are you with the effect of your {treatment/care} at the selected place(s) in Q51b?<br><br>*-Record 99 if don't know/unsure*	0 = Very satisfied<br>1 = Satisfied<br>2 = Neither satisfied nor dissatisfied<br>3 = Dissatisfied<br>4 = Very dissatisfied
Q58b	How satisfied are you with the explanations the {doctor/other health professional} has given you about the results of your {treatment/care} at the selected place(s) in Q51b?<br><br>*-Record 99 if don't know/unsure*	0 = Very satisfied<br>1 = Satisfied<br>2 = Neither satisfied nor dissatisfied<br>3 = Dissatisfied<br>4 = Very dissatisfied

Q59b	The {doctor/other health professional} at the selected place(s) in Q51b was very careful to check everything when examining you.<br><br>*-Record 99 if don't know/unsure*	0 = Strongly agree<br>1 = Agree<br>2 = Not sure<br>3 = Disagree<br>4 = Strongly disagree
Q60b	At the selected place(s) in Q51b, how satisfied were you with the choices you had in decisions affecting your health care?<br><br>*-Record 99 if don't know/unsure*	0 = Very satisfied<br>1 = Satisfied<br>2 = Neither satisfied nor dissatisfied<br>3 = Dissatisfied<br>4 = Very dissatisfied
Q61b	How much of the time did you feel respected by the {doctor/other health professional} at the selected place(s) in Q51b?<br><br>*-Record 99 if don't know/unsure*	0 = All of the time<br>1 = Most of the time<br>2 = About half the time<br>3 = Some of the time<br>4 = None of the time
Q62b	At the selected place(s) in Q51b, the time you had with the {doctor/other health professional} was too short.<br><br>*-Record 99 if don't know/unsure*	0 = Strongly agree<br>1 = Agree<br>2 = Not sure<br>3 = Disagree<br>4 = Strongly disagree
Q63b	Are you satisfied with the care you received in the selected place(s) in Q51b?<br><br>*-Record 99 if don't know/unsure*	0 = Very satisfied<br>1 = Satisfied<br>2 = Neither satisfied nor dissatisfied<br>3 = Dissatisfied<br>4 = Very dissatisfied
Q64b	Would you recommend the selected place(s) in Q51b to others?<br><br>*-Record 99 if don't know/unsure*	1 = Not recommended<br>2 = Recommend with reservations<br>3 = Recommend<br>4 = Highly recommend

Q65b	Have you ever been told by a doctor that you have eyes problems?<br><br>*-Record 99 if don't know/unsure*	0 = No<br>1 = Yes
Q66b	Have you ever been told by a doctor that you have kidney problems?<br><br>*-Record 99 if don't know/unsure*	0 = No<br>1 = Yes
Q67b	Have you ever been told by a doctor that you have lost the sensation of your peripheral membrane, such as foot, hand, arm?<br><br>*-Record 99 if don't know/unsure*	0 = No<br>1 = Yes
Q68b	In **the past 3 months**, besides seeking medical advice or treatment for your diabetes condition, have you sought medical treatment or advice for other illnesses or conditions?<br><br>If No, go to Section 4.<br>If Yes, go to Q24-Q39	0 = No<br>1 = Yes

SECTION 3c: HEALTH CARE UTILIZATION FOR DIABETES AND HYPERTENSION

Q.N	Description and Questions	Response
Q42c	How long have you lived with diabetes?<br><br>*-Record 99 if don't know/unsure and 88 if refuse*<br>*-Less than a year is rounded up to one year*<br>*-Standard rounded up formula is applied.*	________Years
Q43c	Where were you first diagnosed as having diabetes?<br><br>*-Record 99 if don't know and 88 if refuse*	1= National hospital (PP)<br>2= Provincial hospital (RH)<br>3= District hospital (RH)<br>4= Health centre<br>5= Health post<br>6= Provincial rehabilitation centre (PRC) or Community-based rehabilitation (CBR)<br>7= Other public

		8= Private hospital<br>9= Private clinic<br>10= Private pharmacy<br>11= Home/Office of trained health worker/nurse<br>12= Visit of trained health worker/nurse<br>13= Other private medical<br>14= Shop selling drugs/market<br>15= Kru Khmer/ Magician<br>16= Monk/religious leader<br>17= Traditional birth attendant<br>18= Oversee medical service<br>19= MoPoTsyo<br>20= Other; specify:
Q44c	Where did you first seek advice or treatment for diabetes after being diagnosed?<br><br>*-Record 99 if don't know and 88 if refuse*	1= National hospital (PP)<br>2= Provincial hospital (RH)<br>3= District hospital (RH)<br>4= Health centre<br>5= Health post<br>6= Provincial rehabilitation centre (PRC) or Community-based rehabilitation (CBR)<br>7= Other public<br>8= Private hospital<br>9= Private clinic<br>10= Private pharmacy<br>11= Home/Office of trained health worker/nurse<br>12= Visit of trained health worker/nurse<br>13= Other private medical<br>14= Shop selling drugs/market<br>15= Kru Khmer/ Magician<br>16= Monk/religious leader<br>17= Traditional birth attendant<br>18= Oversee medical service<br>19= MoPoTsyo<br>20= Other; specify:
Q45c	How long have you lived with hypertension?<br><br>*-Record 99 if don't know/unsure and 88 if refuse*<br>*-Less than a year is rounded up to one year*<br>*-Standard rounded up formula is applied.*	________Years

Q46c	Where were you first diagnosed as having hypertension?<br><br>*-Record 99 if don't know and 88 if refuse*	1= National hospital (PP)<br>2= Provincial hospital (RH)<br>3= District hospital (RH)<br>4= Health centre<br>5= Health post<br>6= Provincial rehabilitation centre (PRC) or Community-based rehabilitation (CBR)<br>7= Other public<br>8= Private hospital<br>9= Private clinic<br>10= Private pharmacy<br>11= Home/Office of trained health worker/nurse<br>12= Visit of trained health worker/nurse<br>13= Other private medical<br>14= Shop selling drugs/market<br>15= Kru Khmer/ Magician<br>16= Monk/religious leader<br>17= Traditional birth attendant<br>18= Oversee medical service<br>19= MoPoTsyo<br>20= Other; specify:
Q47c	Where did you first seek advice or treatment for hypertension after being diagnosed?<br><br>*-Record 99 if don't know and 88 if refuse*	1= National hospital (PP)<br>2= Provincial hospital (RH)<br>3= District hospital (RH)<br>4= Health centre<br>5= Health post<br>6= Provincial rehabilitation centre (PRC) or Community-based rehabilitation (CBR)<br>7= Other public<br>8= Private hospital<br>9= Private clinic<br>10= Private pharmacy<br>11= Home/Office of trained health worker/nurse<br>12= Visit of trained health worker/nurse<br>13= Other private medical<br>14= Shop selling drugs/market<br>15= Kru Khmer/ Magician<br>16= Monk/religious leader<br>17= Traditional birth attendant<br>18= Oversee medical service<br>19= MoPoTsyo

		20= Other; specify:
Q48c	Did you go to other places for follow up treatment/care for your hypertensive and diabetes conditions?<br><br>*-Record 88 if refuse*<br>*-If NO, please skip Q49c*	0 = No<br>1 = Yes
Q49c	If yes to Q48c, where else did you go to get follow up treatment/care for both conditions?	1= National hospital (PP)<br>2= Provincial hospital (RH)<br>3= District hospital (RH)<br>4= Health centre<br>5= Health post<br>6= Provincial rehabilitation centre (PRC) or Community-based rehabilitation (CBR)<br>7= Other public<br>8= Private hospital<br>9= Private clinic<br>10= Private pharmacy<br>11= Home/Office of trained health worker/nurse<br>12= Visit of trained health worker/nurse<br>13= Other private medical<br>14= Shop selling drugs/market<br>15= Kru Khmer/ Magician<br>16= Monk/religious leader<br>17= Traditional birth attendant<br>18= Oversee medical service<br>19= MoPoTsyo<br>20= Other; specify:
Q50c	Did you get treatment/care for both conditions in the past 12 months?<br><br>*-Record 88 if refuse*<br>*-If NO, please skip Q51c-68c*	0 = No<br>1 = Yes
Q51c	Are you currently receiving any of the following treatment/advices for both conditions prescribed by a doctor or other health care worker?<br><br>Q. 51c.1. Insulin [____]<br>Q. 51c.2. Drugs (medication) that you have taken in the past two weeks [____]<br>Q. 51c.3. Special prescribed diet [____]	0 = No<br>1= Yes

	Q. 51c.4. Advice or treatment to lose weight [____]<br>Q. 51c.5. Advice or treatment to stop smoking [____]<br>Q. 51c.6. Advice to reduce salt intake [____]<br>Q. 51c.7. Advice to start or do more physical exercise [____]<br>Q. 51c.8. Advice to stop drinking alcohol [____]	
Q52c	Have you had your blood glucose measured in the past 12 months?<br><br>*-Record 99 if don't know and 88 if refuse*	0 = No<br>1= Yes
Q53c	Have you had your HbA1c tested in the past 12 months?<br><br>*-Record 99 if don't know and 88 if refuse*	0 = No<br>1= Yes
Q54c	Have you had your blood cholesterol measured in the past 12 months?<br><br>*-Record 99 if don't know and 88 if refuse*	0 = No<br>1= Yes
Q55c	Where did you seek medical advice or treatment for your conditions in **the past 3 months?**<br><br>***-More than one answer can be selected****.*<br>*-Data collectors can use probes to help respondents determine the types of health facilities in the Response Column.*<br>*-Record 99 if don't know and 88 if refuse*	1= National hospital (PP)<br>2= Provincial hospital (RH)<br>3= District hospital (RH)<br>4= Health centre<br>5= Health post<br>6= Provincial rehabilitation centre (PRC) or Community-based rehabilitation (CBR)<br>7= Other public<br>8= Private hospital<br>9= Private clinic<br>10= Private pharmacy<br>11= Home/Office of trained health worker/nurse<br>12= Visit of trained health worker/nurse<br>13= Other private medical

		14= Shop selling drugs/market<br>15= Kru Khmer/ Magician<br>16= Monk/religious leader<br>17= Traditional birth attendant<br>18= Oversee medical service<br>19= MoPoTsyo<br>20= Other; specify:
From Q56c-Q68c, it is a set of questions that are asked following choices selected in Q55c. If 2 or 3 choices were selected in Q55c, Q56c-Q68c would appear 2 or 3 times, accordingly.		
Q56c	How many times did you visit the selected place**(s)** in Q55c in the past three months?	_______ times
Q57c	How much in total was spent on the treatment at the selected place(s) in Q55c?<br><br>*-Record 99 if don't know and 88 if refuse*	0 = free/no cost<br>1 = in kind<br>2 = ________Riels OR<br>________ USD
Q58c	How did you pay for the treatment cost at the selected place(s) in Q55c?<br><br>*-Record 99 if don't know and 88 if refuse*	1= Health Equity Fund<br>2= Voucher<br>3= Fee Exemption<br>4= NGO<br>5= National Social Security Fund<br>6= Community-Based Health Insurance<br>7= Health Insurance through Employer<br>8= Other Privately Purchased Commercial Health Insurance<br>9= Wage/income<br>10= Loan/ Ton Tin<br>11= Sale of Assets<br>12= Gift from Relative<br>13= Savings<br>14= Other

Q59c	How much in total was spent on transport to go to and return from the selected place(s) in Q55c?<br><br>*-Record 99 if don't know and 88 if refuse*	0 = free/no cost<br>1 = in kind<br>2 = ________Riels OR<br>________ USD
Q60c	On average how many hours do you spend to get treatment/advices from the selected place(s) in Q55c?<br><br>*-Record 99 if don't know and 88 if refuse*	________Hours
Q61c	How satisfied are you with the effect of your {treatment/care} at the selected place(s) in Q55c?<br><br>*-Record 99 if don't know/unsure*	0 = Very satisfied<br>1 = Satisfied<br>2 = Neither satisfied nor dissatisfied<br>3 = Dissatisfied<br>4 = Very dissatisfied
Q62c	How satisfied are you with the explanations the {doctor/other health professional} has given you about the results of your {treatment/care} at the selected place(s) in Q55c?<br><br>*-Record 99 if don't know/unsure*	0 = Very satisfied<br>1 = Satisfied<br>2 = Neither satisfied nor dissatisfied<br>3 = Dissatisfied<br>4 = Very dissatisfied
Q63c	The {doctor/other health professional} at the selected place(s) in Q55c was very careful to check everything when examining you.<br><br>*-Record 99 if don't know/unsure*	0 = Strongly agree<br>1 = Agree<br>2 = Not sure<br>3 = Disagree<br>4 = Strongly disagree
Q64c	At the selected place(s) in Q55c, how satisfied were you with the choices you had in decisions affecting your health care?<br><br>*-Record 99 if don't know/unsure*	0 = Very satisfied<br>1 = Satisfied<br>2 = Neither satisfied nor dissatisfied<br>3 = Dissatisfied<br>4 = Very dissatisfied

Q65c	How much of the time did you feel respected by the {doctor/other health professional} at the selected place(s) in Q55c?<br><br>*-Record 99 if don't know/unsure*	0 = All of the time<br>1 = Most of the time<br>2 = About half the time<br>3 = Some of the time<br>4 = None of the time
Q66c	At the selected place(s) in Q55c, the time you had with the {doctor/other health professional} was too short.<br><br>*-Record 99 if don't know/unsure*	0 = Strongly agree<br>1 = Agree<br>2 = Not sure<br>3 = Disagree<br>4 = Strongly disagree
Q67c	Are you satisfied with the care you received in the selected place(s) in Q55c?<br><br>*-Record 99 if don't know/unsure*	0 = Very satisfied<br>1 = Satisfied<br>2 = Neither satisfied nor dissatisfied<br>3 = Dissatisfied<br>4 = Very dissatisfied
Q68c	Would you recommend the selected place(s) in Q55c to others?<br><br>*-Record 99 if don't know/unsure*	1 = Not recommended<br>2 = Recommend with reservations<br>3 = Recommend<br>4 = Highly recommend
Q69c	Have you ever been told by a doctor that you have eyes problems?<br><br>*-Record 99 if don't know/unsure*	0 = No<br>1 = Yes
Q70c	Have you ever been told by a doctor that you have kidney problems?<br><br>*-Record 99 if don't know/unsure*	0 = No<br>1 = Yes

Q71c	Have you ever been told by a doctor that you have lost the sensation of your peripheral membrane, such as foot, hand, arm?<br><br>*-Record 99 if don't know/unsure*	0 = No<br>1 = Yes
Q72c	In **the past 3 months**, besides seeking medical advice or treatment for your hypertensive and diabetes condition, have you sought medical treatment or advice for other illnesses or conditions?<br><br>If No, go to Section 4.<br>If Yes, go to Q24-Q39	0 = No<br>1 = Yes

SECTION 4: SOCIAL SUPPORT

We would like to know if someone from your family (spouse, children, nephew, grandchildren, sibling) provide you support in general. If you need it, how often is **someone from your family** available to:

**Q.N**	**Description and Questions**	**Response**
Q73	have good time with	1. None of the time<br>2. A little of the time<br>3. Some of the time<br>4. Most of the time<br>5. All of the time
Q74	turn to for suggestions about how to deal with a personal problem	1. None of the time<br>2. A little of the time<br>3. Some of the time<br>4. Most of the time<br>5. All of the time
Q75	understand your problems	1. None of the time<br>2. A little of the time<br>3. Some of the time<br>4. Most of the time<br>5. All of the time
Q76	love and make you feel wanted	1. None of the time<br>2. A little of the time<br>3. Some of the time<br>4. Most of the time<br>5. All of the time
Q77	help you if you were confined to bed	1. None of the time<br>2. A little of the time<br>3. Some of the time<br>4. Most of the time<br>5. All of the time

Q78	take you to the doctor if you needed it	1. None of the time<br>2. A little of the time<br>3. Some of the time<br>4. Most of the time<br>5. All of the time
Q79	prepare your meals if you were unable to do it yourself	1. None of the time<br>2. A little of the time<br>3. Some of the time<br>4. Most of the time<br>5. All of the time
Q80	help with daily chores if you were sick	1. None of the time<br>2. A little of the time<br>3. Some of the time<br>4. Most of the time<br>5. All of the time

SECTION 5: LIFESTYLE OR BEHAVIOUR MEASURE

**Q.N**	**Description and Questions**	**Response**
	**Tobacco Use**	
Q81	Do you currently smoke any tobacco products, such as cigarettes, cigars or pipes?<br><br>*-Record 88 if refuse to answer or 99 if don't know*<br><br>**USE SHOWCARD**	0 = No<br>1 = Yes
Q82	Have you smoked a cigarette—even one puff—during the past SEVEN DAYS?<br><br>*Record 88 if refuse to answer or 99 if don't know*<br>**USE SHOWCARD**<br><br>If No, skip Q82a.	0 = No<br>1 = Yes.
Q83	If yes, how many cigarettes did you smoke on average per day?	Number of cigarettes:_____
	**Alcohol Use**	

Q84	Have you ever consumed any alcohol such as beer, spirits or fermented palm juices?<br>**USE SHOWCARD**<br><br>*- Record 88 if refuse to answer or 99 if don't know*	0 = No<br>1 = Yes
Q85	During the PAST MONTH, how frequently have you had at least one standard alcoholic drink?<br>*A drink of alcohol is defined as: one 12-oz can or bottle of beer, one 4-oz glass of wine, one 12-oz can or bottle of wine cooler, 1 mixed drink or cocktail, or 1 shot of hard liquor.*<br>**USE SHOWCARD**<br><br>*If choosing 7, skip Q86*	1 = Daily<br>2 = 5-6 days per week<br>3 = 2-4 days per week<br>4 = 1-2 days per week<br>5 = 1-3 days per month<br>6 = Occasionally, usually less than once a month<br>7 = Never
Q86	What is the largest number of drinks that you've had on any given days (a single occasion) within the last month?<br><br>*(data collector need to calculate the number of drink by counting a drink of alcohol is one 12-oz can or bottle of beer, one 4-oz glass of wine, one 12-oz can or bottle of wine cooler, 1 mixed drink or cocktail, or 1 shot of hard liquor.*	_____ number of drinks
Q87	**During the past 7 days**, when you drink alcohol, how many days per week do you drink alcohol?<br><br>*(Write 0 if they do not drink in the past 7 days)*	_____ number of days
Q88	On a typical day of the past 7 days, that you drink alcohol, h**ow many drinks do you have on a typical day?**<br><br>*(Write 0 if they do not drink in the past 7 days)*	_____number of drinks
**Diet**<br>***A typical week*** *means a "normal" week when the diet is not affected by cultural, religious, or other events.*		

Q89	In the typical week, on how many days do you eat fruit?<br><br>*- Record 88 if refuse to answer or 99 if don't know*<br>**- USE SHOWCARD** *to show examples of fruit*	_______ days
Q90	In a typical week, on how many days do you eat vegetables?<br><br>**- USE SHOWCARD** *to show examples of vegetable (Tubers such as potatoes and cassava should not be included)*<br><br>*- Record 88 if refuse to answer or 99 if don't know*	_______ days
Q91	In a typical week, how many days contain fried vegetables?<br><br>*- Record 88 if refuse to answer or 99 if don't know*	______ days
Q92	In a typical week, how many days do you eat deep fried foods, snacks or fast foods?<br><br>**USE SHOWCARD**<br>*- Record 88 if refuse to answer or 99 if don't know*	_______ days
Q93	In a typical week, on how many days do you drink sugar-sweetened beverages (such as sodas, and other non-carbonated commercially prepared fruit drinks, highly sweetened tea, coffee with condensed milk)?<br>**USE SHOWCARD**<br>*- Record 88 if refuse to answer or 99 if don't know*	_______days
Q94	What type of oil or fat is most often used for meal preparation in your household?<br><br>-Select only one appropriate response.<br>**USE SHOWCARD**<br>*-Record 88 if refuse to answer*<br>*-Record 99 if don't know*	1 = Vegetable Oil<br>2 = Lard or Suet<br>3 = Butter or Ghee<br>4 = Margarine<br>5= Other (specify:)<br>6 = None in particular<br>7 = None used

Q95	How often do you add salt or a salty sauce such as soya sauce or fish sauce to your food right before you eat or as you are eating it?<br>**USE SHOWCARD**<br><br>*-Record 99 if don't know*	1 = Always<br>2 = Often<br>3 = Sometimes<br>4 = Rarely<br>5 = Always<br>6 = Never
Q96	How often is salt, salty seasoning or a salty sauce in cooking or preparing foods in your household?<br><br>**USE SHOWCARD**<br>*-Record 99 if don't know*	1 = Always<br>2 = Often<br>3 = Sometimes<br>4 = Rarely<br>5 = Always<br>6 = Never
Q97	How often do you eat processed food high in salt? By processed food high in salt, I mean food that have been altered from their natural state such as salted fish, salted meat, salted egg, instant noodles, fermented fish (prahok), ba ok, mam, kapik, packaged salty snacks, canned salty food including pickles and preserves, salty food prepared at a fast food restaurant, cheese, bacon and processed meat)<br><br>**USE SHOWCARD**	1 = Always<br>2 = Often<br>3 = Sometimes<br>4 = Rarely<br>5 = Always<br>6 = Never
**PHYSICAL ACTIVITY**		
Q98	On how many of the last SEVEN DAYS did you do **vigorous activities** for **at least 15 minutes**, such as cycling uphill or at fast pace; swimming laps; carrying heavy loads; shovelling or digging; jogging; running or a sport?<br>**USE SHOWCARD** *Record 99 if don't know and 88 if refuse*	Number of days └─┴─┘
Q99	On how many of the last SEVEN DAYS did you do **moderate activities** for **at least 30 minutes**, such as recreational swimming; gardening; heavy cleaning such as washing windows, vacuuming, sweeping or mopping; brisk walking; biking at moderate pace; etc.?<br>**USE SHOWCARD**<br>*Record 99 if don't know and 88 if refuse*	Number of days └─┴─┘

SECTION 6: DIABETES AND HYPERTENSION KNOWLEDGE
(Asking known patients only)

**Q.N**	**Description and Questions**	**Response**
	**Diabetes Knowledge**	
Q100	Eating too much sugar and other sweet foods is a cause of diabetes.<br><br>*Record 99 if don't know and 88 if refuse*	1 = False<br>2 = True
Q101	The usual cause of diabetes is lack of effective insulin in the body.<br><br>*hint: insulin "the hormone which is responsible to regulate the glucose/sugar in the blood and control the normality of blood glucose"*<br><br>*Record 99 if don't know and 88 if refuse*	1 = False<br>2 = True
Q102	Diabetes is caused by failure of the kidneys to keep sugar out of the urine.<br><br>*Record 99 if don't know and 88 if refuse*	1 = False<br>2 = True
Q103	Kidneys produce insulin.<br><br>*hint: insulin "the hormone which is responsible to regulate the glucose/sugar in the blood and control the normality of blood glucose"*<br><br>*Record 99 if don't know and 88 if refuse*	1 = False<br>2 = True
Q104	In untreated diabetes, the amount of sugar in the blood usually increases.<br><br>*Record 99 if don't know and 88 if refuse*	1 = False<br>2 = True
Q105	If I am diabetic, my children have a higher chance of being diabetic.<br><br>*Record 99 if don't know and 88 if refuse*	1 = False<br>2 = True
Q106	Diabetes can be cured.<br><br>*Record 99 if don't know and 88 if refuse*	1 = False<br>2 = True
Q107	A fasting blood sugar level of 210 is too high.<br>Hint: Show a photo of On-Call plus<br><br>*Record 99 if don't know and 88 if refuse*	1 = False<br>2 = True
Q108	The best way to check my diabetes is by testing my urine.<br><br>*Record 99 if don't know and 88 if refuse*	1 = False<br>2 = True

Q109	Regular exercise will increase the need for insulin or other diabetic medication.<br><br>*Record 99 if don't know and 88 if refuse*	1 = False<br>2 = True
Q110	There are two main types of diabetics: Type 1 (insulin dependent) and Type 2 (noninsulin dependent).<br>*Hint: diabetes "type 1" occurs on any age (mostly young people) and "type 2" occur mostly on adult*<br><br>*Record 99 if don't know and 88 if refuse*	1 = False<br>2 = True
Q111	An insulin reaction is caused by too much food.<br><br>*Record 99 if don't know and 88 if refuse*	1 = False<br>2 = True
Q112	Medication is more important than diet and exercise to control my diabetes.<br><br>*Record 99 if don't know and 88 if refuse*	1 = False<br>2 = True
Q113	Diabetes often causes poor circulation.<br><br>*Record 99 if don't know and 88 if refuse*	1 = False<br>2 = True
Q114	Cuts and abrasions on diabetes heal more slowly.<br><br>*Record 99 if don't know and 88 if refuse*	1 = False<br>2 = True
Q115	Diabetes should take extra care when cutting their toenails.<br><br>*Record 99 if don't know and 88 if refuse*	1 = False<br>2 = True
Q116	A person with diabetes should cleanse a cut with iodine and alcohol.<br><br>*Record 99 if don't know and 88 if refuse*	1 = False<br>2 = True
Q117	The way I prepare my food is as important as the foods I eat.<br><br>*Record 99 if don't know and 88 if refuse*	1 = False<br>2 = True
Q118	Diabetes can cause loss of feeling in my hands, fingers and feet.<br><br>*Record 99 if don't know and 88 if refuse*	1 = False<br>2 = True
Q119	Shaking and sweating are signs of low blood sugar.<br><br>*Record 99 if don't know and 88 if refuse*	1 = False<br>2 = True
Q120	Frequent urination and thirst are signs of low blood sugar.<br><br>*Record 99 if don't know and 88 if refuse*	1 = False<br>2 = True

Q121	Tight elastic hose or socks are not bad for diabetics.<br><br>*Record 99 if don't know and 88 if refuse*	1 = False<br>2 = True
Q122	A diabetic diet consists mostly of special foods.<br><br>*Record 99 if don't know and 88 if refuse*	1 = False<br>2 = True
	**Hypertension Knowledge**	
Q123	Increased diastolic blood pressure also indicates increased blood pressure.<br>*Hint: Show a photo of OMRON machine*<br><br>*Record 99 if don't know and 88 if refuse*	1 = False<br>2 = True
Q124	High diastolic or systolic blood pressure indicates increased blood pressure.<br>*Hint: Show a photo of OMRON machine*<br><br>*Record 99 if don't know and 88 if refuse*	1 = False<br>2 = True
Q125	Drugs for increased blood pressure must be taken every day.<br><br>*Record 99 if don't know and 88 if refuse*	1 = False<br>2 = True
Q126	Individuals with increased blood pressure must take their medication only when they feel ill.<br><br>*Record 99 if don't know and 88 if refuse*	1 = False<br>2 = True
Q127	Individuals with increased blood pressure must take their medication throughout their life.<br><br>*Record 99 if don't know and 88 if refuse*	1 = False<br>2 = True
Q128	Individuals with increased blood pressure must take their medication in a manner that makes them feel good.<br><br>*Record 99 if don't know and 88 if refuse*	1 = False<br>2 = True
Q129	If the medication for increased blood pressure can control blood pressure, there is no need to change lifestyles.<br><br>*Record 99 if don't know and 88 if refuse*	1 = False<br>2 = True
Q130	Increased blood pressure is the result of aging, so treatment is unnecessary.<br><br>*Record 99 if don't know and 88 if refuse*	1 = False<br>2 = True

Q131	If individuals with increased blood pressure change their lifestyles, there is no need or treatment.<br><br>*Record 99 if don't know and 88 if refuse*	1 = False<br>2 = True
Q132	Individuals with increased blood pressure can eat salty foods as long as they take their drugs regularly.<br><br>*Record 99 if don't know and 88 if refuse*	1 = False<br>2 = True
Q133	Individuals with increased blood pressure can drink alcoholic beverages.<br><br>*Record 99 if don't know and 88 if refuse*	1 = False<br>2 = True
Q134	Individuals with increased blood pressure must not smoke.<br><br>*Record 99 if don't know and 88 if refuse*	1 = False<br>2 = True
Q135	Individuals with increased blood pressure must eat fruits and vegetables frequently.<br><br>*Record 99 if don't know and 88 if refuse*	1 = False<br>2 = True
Q136	For individuals with increased blood pressure, the best cooking method is frying.<br><br>*Record 99 if don't know and 88 if refuse*	1 = False<br>2 = True
Q137	For individuals with increased blood pressure, the best cooking method is boiling or grilling.<br><br>*Record 99 if don't know and 88 if refuse*	1 = False<br>2 = True
Q138	The best type of meat for individuals with increased blood pressure is white meat.<br><br>*Record 99 if don't know and 88 if refuse*	1 = False<br>2 = True
Q139	The best type of meat for individuals with increased blood pressure is red meat.<br><br>*Record 99 if don't know and 88 if refuse*	1 = False<br>2 = True
Q140	Increased blood pressure can cause premature death if left untreated.<br><br>*Record 99 if don't know and 88 if refuse*	1 = False<br>2 = True
Q141	Increased blood pressure can cause heart diseases, such as heart attack, if left untreated.<br><br>*Record 99 if don't know and 88 if refuse*	1 = False<br>2 = True
Q142	Increased blood pressure can cause strokes, if left untreated.<br><br>*Record 99 if don't know and 88 if refuse*	1 = False<br>2 = True

Q143	Increased blood pressure can cause kidney failure, if left untreated.<br><br>*Record 99 if don't know and 88 if refuse*	1 = False<br>2 = True
Q144	Increased blood pressure can cause visual disturbances, if left untreated.<br><br>*Record 99 if don't know and 88 if refuse*	1 = False<br>2 = True

SECTION 7a: MEDICATION ADHERENCE FOR HYPERTENSION
(Asking known hypertension only)

**Q.N**	**Description and Questions**	**Response**
Can you rate the frequency with which you engaged in each of the below aspects?		
Q145 a	What type of medicine are you currently using for hypertension treatment?<br><br>Hint: *this section is to assess medication adherence for those who currently administer pill (1 and 2). For patients using traditional medicine or herbal medicine (choose 3 and 4), skip this part.*	1. Pill<br>2. Combination between pills and traditional medicine<br>3. Use only traditional medicine (herbal)<br>4. Other (please specify)
Q146 a	Forget to take your hypertension medicines<br>*Record 99 if don't know and 88 if refuse*	1. Very often<br>2. Often<br>3. Sometimes<br>4. Rarely<br>5. Never
Q147 a	Alter the dose of hypertension medicines<br>*Record 99 if don't know and 88 if refuse*	1. Very often<br>2. Often<br>3. Sometimes<br>4. Rarely<br>5. Never
Q148 a	Stop the hypertension medicine for a while<br>*Record 99 if don't know and 88 if refuse*	1. Very often<br>2. Often<br>3. Sometimes<br>4. Rarely<br>5. Never
Q149 a	Miss out on a dose of your hypertension medicine<br>*Record 99 if don't know and 88 if refuse*	1. Very often<br>2. Often<br>3. Sometimes<br>4. Rarely<br>5. Never
Q150 a	Take less medicines than instructed<br>*Record 99 if don't know and 88 if refuse*	1. Very often<br>2. Often

		3. Sometimes<br>4. Rarely<br>5. Never

SECTION 7b: MEDICATION ADHERENCE FOR DIABETES
(Asking known diabetes only)

Q.N	Description and Questions	Response
Can you rate the frequency with which you engaged in each of the below aspects?		
Q145 b	What type of medicine are you currently using for diabetes treatment?<br><br>Hint*: this section is to assess medication adherence for those who currently administer pill or injection (insulin) (1, 2 and 3). For patients using traditional medicine or herbal medicine (choose 4 and 5), skip this part.*	1. Pill<br>2. Injection (Insulin)<br>3. Combination between pills and traditional medicine<br>4. Use only traditional medicine (herbal)<br>5. Other (please specify)
Q146 b	Forget to take your diabetes medicines<br>*Record 99 if don't know and 88 if refuse*	1. Very often<br>2. Often<br>3. Sometimes<br>4. Rarely<br>5. Never
Q147 b	Alter the dose of your diabetes medicines<br>*Record 99 if don't know and 88 if refuse*	1. Very often<br>2. Often<br>3. Sometimes<br>4. Rarely<br>5. Never
Q148 b	Stop the diabetes medicine for a while<br>*Record 99 if don't know and 88 if refuse*	1. Very often<br>2. Often<br>3. Sometimes<br>4. Rarely<br>5. Never
Q149 b	Miss out on a dose of the your diabetes medicine<br>*Record 99 if don't know and 88 if refuse*	1. Very often<br>2. Often<br>3. Sometimes<br>4. Rarely<br>5. Never
Q150 b	Take less medicines than instructed<br>*Record 99 if don't know and 88 if refuse*	1. Very often<br>2. Often

		3. Sometimes<br>4. Rarely<br>5. Never

Known patients with both hypertension and diabetes will have to go through Section 07a and 07b.

SECTION 8: SELF-MANAGEMENT SUPPORT

(Asking known patients only)

Q.N	Description and Questions	Response
	**A. Family**<br>*We would like to know how often anyone from your family offer you self-management support in the following aspects:*	
Q151	Eat healthy food with you<br><br>*Record 99 if don't know and 88 if refuse*	1. Never<br>2. less than once a week<br>3. once a week<br>4. more than once a week
Q152	Eat unhealthy food with you<br><br>*Record 99 if don't know/not applicable and 88 if refuse*	1. Never<br>2. less than once a week<br>3. once a week<br>4. more than once a week
Q153	Encourage you to stick with your healthy diet<br><br>*Record 99 if don't know/not applicable and 88 if refuse*	1. Never<br>2. less than once a week<br>3. once a week<br>4. more than once a week
Q154	Encourage you to eat unhealthy food (such as junk food, fried food etc)<br><br>*Record 99 if don't know/not applicable and 88 if refuse*	1. Never<br>2. less than once a week<br>3. once a week<br>4. more than once a week
Q155	Exercise with you<br><br>*Record 99 if don't know/not applicable and 88 if refuse*	1. Never<br>2. less than once a week<br>3. once a week<br>4. more than once a week
Q156	Encourage you to do exercise	1. Never<br>2. less than once a week

	Record 99 if don't know/not applicable and 88 if refuse	3. once a week<br>4. more than once a week
Q157	Help you take your medication correctly (explain based on prescription)<br><br>*Record 99 if don't know/not applicable and 88 if refuse*	1. Never<br>2. less than once a week<br>3. once a week<br>4. more than once a week
Q158	Remind you to take your medications regularly<br><br>*Record 99 if don't know/not applicable and 88 if refuse*	1. Never<br>2. less than once a week<br>3. once a week<br>4. more than once a week
Q159	Encourage you to skip your medications<br><br>*Record 99 if don't know/not applicable and 88 if refuse*	1. Never<br>2. less than once a week<br>3. once a week<br>4. more than once a week
Q160	Help you to self-monitor of your blood pressure or blood glucose<br><br>*Record 99 if don't know/not applicable and 88 if refuse*	1. Never<br>2. less than once a week<br>3. once a week<br>4. more than once a week
Q161	Remind you to self-monitor your blood pressure or blood glucose<br><br>*Record 99 if don't know/not applicable and 88 if refuse*	1. Never<br>2. less than once a week<br>3. once a week<br>4. more than once a week
Q162	Remind you of your follow-up schedule at the health facility<br><br>*Record 99 if don't know/not applicable and 88 if refuse*	1. Never<br>2. less than once a week<br>3. once a week<br>4. more than once a week
Q163	Encourage you to visit the facility regularly for follow-up treatment<br><br>*Record 99 if don't know/not applicable and 88 if refuse*	1. Never<br>2. less than once a week<br>3. once a week<br>4. more than once a week
Q164	Bring you to the health facilities for follow-up visit	1. Never<br>2. less than once a week<br>3. once a week

	Record 99 if don't know/not applicable and 88 if refuse	4. more than once a week
**B. Non-family support**<br>*We would like to know how often anyone from non-family such as friend, neighbour, colleague or fellow patient (not peer educator) offer you self-management support in the following aspects:*		
Q165	Eat healthy food with you<br><br>*Record 99 if don't know/not applicable and 88 if refuse*	1. Never<br>2. less than once a week<br>3. once a week<br>4. more than once a week
Q166	Eat unhealthy food with you<br><br>*Record 99 if don't know/not applicable and 88 if refuse*	1. Never<br>2. less than once a week<br>3. once a week<br>4. more than once a week
Q167	Encourage you to stick with your healthy diet<br><br>*Record 99 if don't know/not applicable and 88 if refuse*	1. Never<br>2. less than once a week<br>3. once a week<br>4. more than once a week
Q168	Encourage you to eat unhealthy food (such as junk food, fried food etc.)<br><br>*Record 99 if don't know/not applicable and 88 if refuse*	1. Never<br>2. less than once a week<br>3. once a week<br>4. more than once a week
Q169	Exercise with you<br><br>*Record 99 if don't know/not applicable and 88 if refuse*	1. Never<br>2. less than once a week<br>3. once a week<br>4. more than once a week
Q170	Encourage you to do exercise<br><br>*Record 99 if don't know/not applicable and 88 if refuse*	1. Never<br>2. less than once a week<br>3. once a week<br>4. more than once a week
Q171	Help you take your medication correctly (explain based on prescription)<br><br>*Record 99 if don't know/not applicable and 88 if refuse*	1. Never<br>2. less than once a week<br>3. once a week<br>4. more than once a week
Q172	Remind you to take your medications regularly	1. Never

	Record 99 if don't know/not applicable and 88 if refuse	2. less than once a week<br>3. once a week<br>4. more than once a week
Q173	Encourage you to skip your medications<br><br>*Record 99 if don't know/not applicable and 88 if refuse*	1. Never<br>2. less than once a week<br>3. once a week<br>4. more than once a week
Q174	Help you to self-monitor of your blood pressure or blood glucose<br><br>*Record 99 if don't know/not applicable and 88 if refuse*	1. Never<br>2. less than once a week<br>3. once a week<br>4. more than once a week
Q175	Remind you to self-monitor your blood pressure or blood glucose<br><br>*Record 99 if don't know/not applicable and 88 if refuse*	1. Never<br>2. less than once a week<br>3. once a week<br>4. more than once a week
Q176	Remind you of your follow-up schedule at the health facility<br><br>*Record 99 if don't know/not applicable and 88 if refuse*	1. Never<br>2. less than once a week<br>3. once a week<br>4. more than once a week
Q177	Encourage you to visit the facility regularly for follow-up treatment<br><br>*Record 99 if don't know/not applicable and 88 if refuse*	1. Never<br>2. less than once a week<br>3. once a week<br>4. more than once a week
Q178	Bring you to the health facilities for follow-up visit<br><br>*Record 99 if don't know/not applicable and 88 if refuse*	1. Never<br>2. less than once a week<br>3. once a week<br>4. more than once a week
**C. Community Organization**<br>*This section refers to Village Health Support Group or other people who work closely in the village to provide health-related activities* ***excluding*** *Peer Educator Network.*		
Q179	Are there anyone from community organization provide you support with your conditions?<br><br>*If No, go to Part D of Peer Educator*	0 = No<br>1= Yes

If you need it, how often is someone from community organization or community health worker available to:		
Q180	Give you suggestions about how to deal with health problem<br><br>*Record 99 if don't know/not applicable and 88 if refuse*	1. Never<br>2. less than once a week<br>3. once a week<br>4. more than once a week
Q181	Provide you guidance on available health service at the public health facilities<br>*Record 99 if don't know/not applicable and 88 if refuse*	1. Never<br>2. less than once a week<br>3. once a week<br>4. more than once a week
Q182	Bring you to the health facilities if you need help<br><br>*Record 99 if don't know/not applicable and 88 if refuse*	1. Never<br>2. less than once a week<br>3. once a week<br>4. more than once a week
Q183	Introduce you the healthy diet<br><br>*Record 99 if don't know/not applicable and 88 if refuse*	1. Never<br>2. less than once a week<br>3. once a week<br>4. more than once a week
Q184	Encourage you to eat healthy food<br><br>*Record 99 if don't know/not applicable and 88 if refuse*	1. Never<br>2. less than once a week<br>3. once a week<br>4. more than once a week
Q185	Encourage you to eat unhealthy food<br><br>*Give example of unhealthy food*<br><br>*Record 99 if don't know/not applicable and 88 if refuse*	1. Never<br>2. less than once a week<br>3. once a week<br>4. more than once a week
Q186	Introduce you how to be physically active<br><br>*Record 99 if don't know/not applicable and 88 if refuse*	1. Never<br>2. less than once a week<br>3. once a week<br>4. more than once a week
Q187	Encourage you to do exercise<br><br>*Record 99 if don't know/not applicable and 88 if refuse*	1. Never<br>2. less than once a week<br>3. once a week<br>4. more than once a week

Q188	Help you to take your medication correctly (explain based on prescription)<br><br>*Record 99 if don't know/not applicable and 88 if refuse*	1. Never<br>2. less than once a week<br>3. once a week<br>4. more than once a week
Q189	Remind you to take your medications regularly<br><br>*Record 99 if don't know/not applicable and 88 if refuse*	1. Never<br>2. less than once a week<br>3. once a week<br>4. more than once a week
Q190	Encourage you to skip your medications<br><br>*Record 99 if don't know/not applicable and 88 if refuse*	1. Never<br>2. less than once a week<br>3. once a week<br>4. more than once a week
Q191	Help you to self-monitor of your blood pressure or blood glucose<br><br>*Record 99 if don't know/not applicable and 88 if refuse*	1. Never<br>2. less than once a week<br>3. once a week<br>4. more than once a week
Q192	Encourage you to self-monitor your blood pressure of blood glucose<br><br>*Record 99 if don't know/not applicable and 88 if refuse*	1. Never<br>2. less than once a week<br>3. once a week<br>4. more than once a week
Q193	Remind you of your appointment schedule at the health facility<br><br>*Record 99 if don't know/not applicable and 88 if refuse*	1. Never<br>2. less than once a week<br>3. once a week<br>4. more than once a week
Q194	Encourage you to visit health facility regularly for follow-up appointment<br><br>*Record 99 if don't know/not applicable and 88 if refuse*	1. Never<br>2. less than once a week<br>3. once a week<br>4. more than once a week
Q195	Visit your home for individual health education<br><br>*Record 99 if don't know/not applicable and 88 if refuse*	1. Never<br>2. less than once a week<br>3. once a week<br>4. more than once a week
Q196	Provide group health education on self-management	1. Never<br>2. less than once a week

	Record 99 if don't know/not applicable and 88 if refuse	3. once a week<br>4. more than once a week
Q197	Are you satisfied with the support you received from community health workers?<br><br>*Record 99 if don't know/not applicable and 88 if refuse*	1. Very satisfied<br>2. Satisfied<br>3. neutral<br>4. Dissatisfied<br>5. Satisfied
**D. Peer Educator Network**<br>*(Diabetic patients working for MoPoTsyo)*		
Q198	Is there anyone from **peer educator network** providing you support in general and with your health conditions?<br><br>*If No, go to Section 9.*	0 = No<br>1= Yes
If you need support, how often is ***someone from peer education network*** *available to:*		
Q199	Give you suggestions about how to deal with health problem<br><br>*Record 99 if don't know/not applicable and 88 if refuse*	1. Never<br>2. less than once a week<br>3. once a week<br>4. more than once a week
Q200	Provide you guidance on available health service at the public health facilities<br><br>*Record 99 if don't know/not applicable and 88 if refuse*	1. Never<br>2. less than once a week<br>3. once a week<br>4. more than once a week
Q201	Bring you to the health facilities if you need help<br><br>*Record 99 if don't know/not applicable and 88 if refuse*	1. Never<br>2. less than once a week<br>3. once a week<br>4. more than once a week
Q202	Introduce you the healthy diet<br><br>*Record 99 if don't know/not applicable and 88 if refuse*	1. Never<br>2. less than once a week<br>3. once a week<br>4. more than once a week
Q203	Encourage you to eat healthy<br><br>*Record 99 if don't know/not applicable and 88 if refuse*	1. Never<br>2. less than once a week<br>3. once a week<br>4. more than once a week
Q204	Encourage you to eat unhealthy food	1. Never<br>2. less than once a week

	Record 99 if don't know/not applicable and 88 if refuse	3. once a week<br>4. more than once a week
Q205	Introduce you how to be physically active<br><br>*Record 99 if don't know/not applicable and 88 if refuse*	1. Never<br>2. less than once a week<br>3. once a week<br>4. more than once a week
Q206	Encourage you to do exercise<br><br>*Record 99 if don't know/not applicable and 88 if refuse*	1. Never<br>2. less than once a week<br>3. once a week<br>4. more than once a week
Q207	Help you to take your medication correctly (explain based on prescription)<br><br>*Record 99 if don't know/not applicable and 88 if refuse*	1. Never<br>2. less than once a week<br>3. once a week<br>4. more than once a week
Q208	Remind you to take your medications regularly<br><br>*Record 99 if don't know/not applicable and 88 if refuse*	1. Never<br>2. less than once a week<br>3. once a week<br>4. more than once a week
Q209	Encourage you to skip your medications<br><br>*Record 99 if don't know/not applicable and 88 if refuse*	1. Never<br>2. less than once a week<br>3. once a week<br>4. more than once a week
Q210	Help you to self-monitor of your blood pressure or blood glucose<br><br>*Record 99 if don't know/not applicable and 88 if refuse*	1. Never<br>2. less than once a week<br>3. once a week<br>4. more than once a week
Q211	Encourage you to self-monitor your blood pressure or blood glucose<br><br>*Record 99 if don't know/not applicable and 88 if refuse*	1. Never<br>2. less than once a week<br>3. once a week<br>4. more than once a week
Q212	Remind you of your appointment at the health facility	1. Never<br>2. less than once a week<br>3. once a week

	Record 99 if don't know/not applicable and 88 if refuse	4. more than once a week
Q213	Encourage you to visit health facility regularly for follow-up appointment<br><br>*Record 99 if don't know/not applicable and 88 if refuse*	1. Never<br>2. less than once a week<br>3. once a week<br>4. more than once a week
Q214	Visit your home for individual health education<br><br>*Record 99 if don't know/not applicable and 88 if refuse*	1. Never<br>2. less than once a week<br>3. once a week<br>4. more than once a week
Q215	Provide group health education on self-management<br><br>*Record 99 if don't know/not applicable and 88 if refuse*	1. Never<br>2. less than once a week<br>3. once a week<br>4. more than once a week
Q216	Are you satisfied with the support you received from the peer educator network?<br><br>*Record 99 if don't know/not applicable and 88 if refuse*	1. Very satisfied<br>2. Satisfied<br>3. neutral<br>4. Dissatisfied<br>5. Very dissatisfied

SECTION 9: DECISION MAKING POWER ON DIET
(Asking known patients only)

We want to understand to what extend you have power in food making/consumption in your family.		
Q217	Who is your household's primary grocery shopper?	1. yourself<br>2. spouse<br>3. other in the household
Q218	Whose preference did the shopper accommodating to most of the time?	1. yourself<br>2. spouse<br>3. other in the household
Q219	Who is in your household usually prepares food?	1. yourself<br>2. spouse<br>3. other in the household
Q220	Whose preference did the cook accommodating to most of the time?	1. yourself<br>2. spouse<br>3. other in the household
Q221	How much influence do you have in the decision on what you eat?	1. Somebody else decides for me, I

		never make the decision<br>2. Somebody else decides for me, I rarely make the decision<br>3. Somebody else decides and I sometimes make the decision<br>4. I often make my own decision<br>5. I always make my own decision without any interference

SECTION 10: ANTHROPOMETRIC MEASUREMENTS

Q.N	Description and Questions	Response
Q222	Height	_________Cm
Q223	Weight	_________Kg
Q224	Waist circumference	________ Cm
Q225	Hip circumference	________ Cm

Q226	Blood pressure, reading 1	_______Systolic (mmHg)<br><br>_______Diastolic (mmHg)
Q227	Blood pressure, reading 2	_______Systolic (mmHg)<br><br>_______Diastolic (mmHg)
Q228	Blood pressure, reading 3	_______Systolic (mmHg)<br><br>_______Diastolic (mmHg)

SECTION 11: BIOMARKER MEASUREMENTS

**Q.N**	**Description and Questions**	**Response**
Q229	During the past 10 hours have you had anything to eat or drink other than plain water?<br><br>*-If YES, do not proceed.*	0 = No<br>1 = Yes
Q230	Fasting Blood Glucose	________mg/dl
Q231	HbA1c	_______ mmol/m
Q232	Creatinine	_______<br>mg/dl

[End of Questionnaire]
