# Supplementary material for: Generation of Cascades of Care for Diabetes and Hypertension Care Continuum in Cambodia: Protocol for a Population-Based Survey Protocol
Source: JMIR Res Protoc. 2022 Sep 2;11(9):e36747. doi: 10.2196/36747 (PMC9482065; doi:10.2196/36747)
Supplement: Multimedia Appendix 3 [file resprot_v11i9e36747_app3.pdf]

Multimedia Appendix 3. Sample List of the Eligible Households in Each Village

Village Code PR191	Village/ Commune / District _____/_______/_______	OD __________	Health Centre ____________	Listing team :
				Listed on:
Block: 1	Contact of local authority: _________________		Scheduled on: ________________	Data Collection Team:

Household Code		Name of Household Head	Household Contact Number	Participant in Household Questionnaire	N. of members >= 40	Name of eligible individual (unchangeable)
PR191	01					
PR191	02					
PR191	03					
PR191	04					
PR191	05					
PR191	06					
PR191	07					
PR191	08					
PR191	09					
PR191	10					
PR191	11					
PR191	12					
PR191	13					
PR191	14					
PR191	15					
PR191	16					
PR191	17					
PR191	18					
PR191	19					
PR191	20					
PR191	21					
PR191	22					
PR191	23					
PR191	24					

Note: Participant in household questionnaire can be any household member aged 18 and above and well-informed about the household situation (including the eligible individual).
