# Supplementary material for: Generation of Cascades of Care for Diabetes and Hypertension Care Continuum in Cambodia: Protocol for a Population-Based Survey Protocol
Source: JMIR Res Protoc. 2022 Sep 2;11(9):e36747. doi: 10.2196/36747 (PMC9482065; doi:10.2196/36747)
Supplement: Multimedia Appendix 4 [file resprot_v11i9e36747_app4.pdf]

Multimedia Appendix 4. Sample of Record Book of Anthropometric and Biochemical Measurements of All the Eligible Individuals

Village Code	Village/ Commune / District	Health Centre	Data Collector Group:	Other
PR191	_____/______/______	____________	Collected on:	

Code of eligible individual			Name of eligible individual	Sex	Known condition		FBG (mg/dl)	Blood pressure measurements			Height (cm)	Weight (kg)	Hip circumference (cm)	Waist circumference (cm)
					T2D	HT		First Time (Sys./Dia.) mmHg	Second Time (Sys./Dia.) mmHg	Third Time (Sys./Dia.) mmHg				
PR191	01	01												
PR191	02	01												
PR191	03	01												
PR191	04	01												
PR191	05	01												
PR191	06	01												
PR191	07	01												
PR191	08	01												
PR191	09	01												
PR191	10	01												
PR191	11	01												
PR191	12	01												
PR191	13	01												
PR191	14	01												
PR191	15	01												
PR191	16	01												
PR191	17	01												
PR191	18	01												
PR191	19	01												
PR191	20	01												
PR191	21	01												
PR191	22	01												
PR191	23	01												
PR191	24	01												
