# Supplementary material for: Generation of Cascades of Care for Diabetes and Hypertension Care Continuum in Cambodia: Protocol for a Population-Based Survey Protocol
Source: JMIR Res Protoc. 2022 Sep 2;11(9):e36747. doi: 10.2196/36747 (PMC9482065; doi:10.2196/36747)
Supplement: Multimedia Appendix 5 [file resprot_v11i9e36747_app5.pdf]

Multimedia Appendix 5. Sample Record book of HbA1c and Creatinine for Known T2D Patients or Participants having FBG ≥126 mg/dl

Village Code PR191			Village/ Commune / District _____/______/______	Health Centre ___________		Data Collector : ________________ Collected on: ___________________	
Code of eligible individual			Name of eligible individual	Sex	T2D condition		HbA1c (mmol/m)	Creatinine (mg/dl)
					Known	Suspected		
PR191	01	01						
PR191	02	01						
PR191	03	01						
PR191	04	01						
PR191	05	01						
PR191	06	01						
PR191	07	01						
PR191	08	01						
PR191	09	01						
PR191	10	01						
PR191	11	01						
PR191	12	01						
PR191	13	01						
PR191	14	01						
PR191	15	01						
PR191	16	01						
PR191	17	01						
PR191	18	01						
PR191	19	01						
PR191	20	01						
PR191	21	01						
PR191	22	01						
PR191	23	01						
PR191	24	01						
