# Supplementary material for: Generation of Cascades of Care for Diabetes and Hypertension Care Continuum in Cambodia: Protocol for a Population-Based Survey Protocol
Source: JMIR Res Protoc. 2022 Sep 2;11(9):e36747. doi: 10.2196/36747 (PMC9482065; doi:10.2196/36747)
Supplement: Multimedia Appendix 6 [file resprot_v11i9e36747_app6.pdf]

Multimedia Appendix 6. Identified Explanatory Variables

Explanatory variable	***Classification***	***Questions extracted for analysis***
Socio-demographic variables		
Age groups	(1) 40-49<br>(2) 50-59<br>(3) 60-69<br>(4) 70-79<br>(5) 80 +	How old are you?
Sex	(1) Female<br>(2) Male	Sex of participant
Marital status	(1) Married or living with spouse<br>(2) Widowed or not living with spouse	What is your marital status?
Educational levels	(1) No formal education or less than primary education<br>(2) Primary education<br>(3) Secondary education or above	What is your highest educational level?
Household wealth quintile	(1) 1 (The poorest)<br>(2) 2<br>(3) 3<br>(4) 4<br>(5) 5 (The richest)	Household socio-economic status questions (Principal Component Analysis) [1]
Household size	(1) 1-4<br>(2) 5+	How many household members are there in your household?
Health care utilisation variables		
Health care provider sought in the past 3 months	(1) Public provider<br>(2) Private provider<br>(3) NGOs<br>(4) Others	Where did you seek medical advice or treatment for your condition in the past 3 months?
Satisfaction of care service	(1) Score 0-10 (Very dissatisfied)<br>(2) Score 11-18 (Dissatisfied)<br>(3) Score 19-26 (Satisfied)<br>(4) Score 27-28 (Very satisfied)	Patient satisfaction of health care service set of questions (Short Assessment of Patient Satisfaction Analysis) [2]
Lifestyle variables		
Tobacco smoking	(1) Not currently smoking<br>(2) Currently smoking	Do you currently smoke any tobacco products, such as cigarettes, cigars or pipes?

[1] Abdi H, Williams LJ. Principal component analysis. Wiley interdisciplinary reviews: computational statistics. 2010;2(4):433-59.
[2] Hawthorne G, Sansoni J, Hayes L, Marosszeky N, Sansoni E. Measuring patient satisfaction with health care treatment using the Short Assessment of Patient Satisfaction measure delivered superior and robust satisfaction estimates. Journal of Clinical Epidemiology. 2014;67(5):527-37.

Alcohol consumption	(1) No<br>(2) Yes	Have you ever consumed any alcohol such as beer, spirits or fermented palm juices?
Physical activity	(1) 0<br>(2) 1-3<br>(3) 4-5<br>(4) 6-7	On how many of the last SEVEN DAYS did you do moderate activities for at least 30 minutes, such as recreational swimming; gardening; heavy cleaning such as washing windows, vacuuming, sweeping or mopping; brisk walking; biking at moderate pace; etc.?
Condition and knowledge variables		
Knowledge of T2D	Diabetes Knowledge Questionnaire sum-scale score 0-24	Diabetes Knowledge set of questions [3]
Knowledge of HT	Hypertension Knowledge-Level Scale (score 0-22)<br>(1) Low level of knowledge (score ≤17)<br>(2) High level of knowledge (score 18-22)	Hypertension knowledge set of questions [4]
Duration of T2D	(1) 1 year<br>(2) 2-5 years<br>(3) More than 5 years	How long have you lived with diabetes?
Duration of HT	(1) 1 year<br>(2) 2-5 years<br>(3) More than 5 years	How long have you lived with hypertension?
Medication adherence variables		
Medication adherence for T2D	(1) Low adherers<br>(2) High adherers	Medication adherence set of questions [5]
Medication adherence for HT	(1) Low adherers<br>(2) High adherers	Medication adherence set of questions [5]
Anthropometric variables		
BMI categories	(1) Underweight (BMI <18.5 kg/m^2	Weight measurement / [height measurement]^2

[3] Bukhsh A, Khan TM, Nawaz MS, Ahmed HS, Chan KG, Goh B-H. Association of diabetes knowledge with glycemic control and self-care practices among Pakistani people with type 2 diabetes mellitus. Diabetes, Metabolic Syndrome and Obesity: Targets and Therapy. 2019;12:1409.
[4] Jankowska-Polańska B, Uchmanowicz I, Dudek K, Mazur G. Relationship between patients' knowledge and medication adherence among patients with hypertension. Patient Preference and Adherence. 2016;10:2437.
[5] Chan AHY, Horne R, Hankins M, Chisari C. The medication adherence report scale: a measurement tool for eliciting patients' reports of nonadherence. British Journal of Clinical Pharmacology. 2020.

	(2) Healthy weight (18.5 $kg/m^2 \le$ BMI < 25 kg/m^2<br>(3) Overweight (25 $kg/m^2 \le$ BMI < 30 kg/m^2<br>(3) Obese (BMI ≥ 30 kg/m^2)	
